# Heat Dissipation Mechanisms in Hybrid Superconductor–Semiconductor Devices Revealed by Joule Spectroscopy

**DOI:** 10.1021/acs.nanolett.4c00574

**Published:** 2024-05-21

**Authors:** Ángel Ibabe, Gorm O. Steffensen, Ignacio Casal, Mario Gómez, Thomas Kanne, Jesper Nygård, Alfredo Levy Yeyati, Eduardo J. H. Lee

**Affiliations:** † Departamento de Física de la Materia Condensada, Universidad Autónoma de Madrid, E-28049 Madrid, Spain; ‡ Condensed Matter Physics Center (IFIMAC), Universidad Autónoma de Madrid, E-28049 Madrid, Spain; ¶ Departamento de Física Teórica de la Materia Condensada, Universidad Autónoma de Madrid, E-28049 Madrid, Spain; § Center for Quantum Devices, Niels Bohr Institute, University of Copenhagen, DK-2100 Copenhagen, Denmark; ∥ Instituto Nicolás Cabrera, Universidad Autónoma de Madrid, E-28049 Madrid, Spain

**Keywords:** hybrid superconductor−semiconductor devices, thermal transport, Joule heating, quantum devices, nanowires

## Abstract

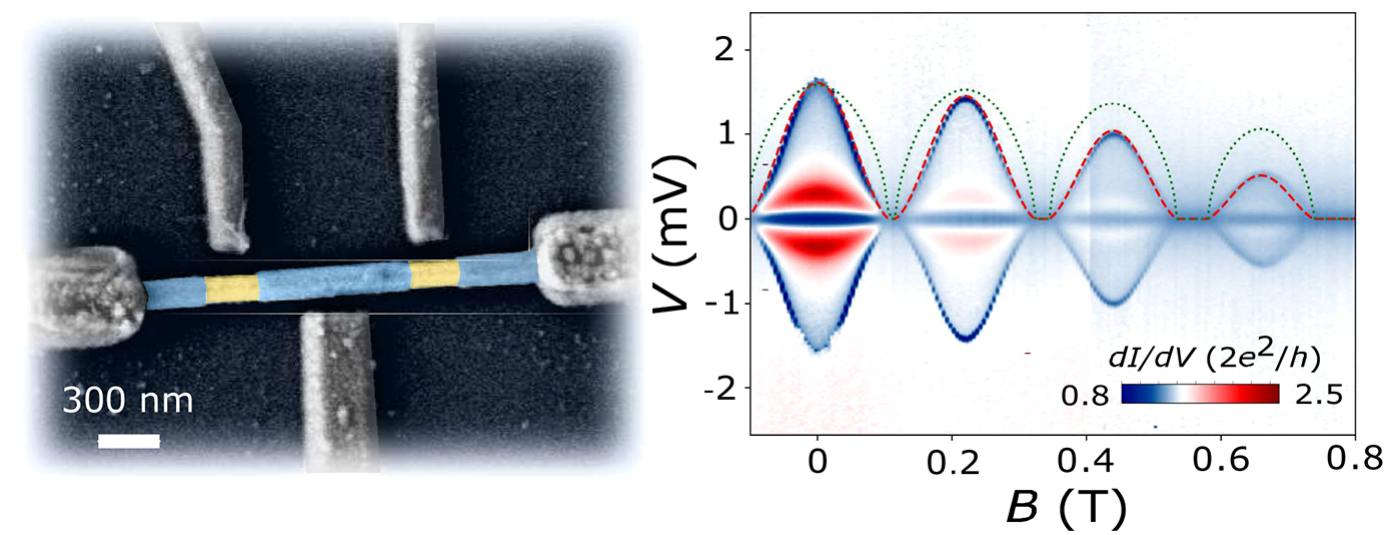

Understanding heating and cooling mechanisms in mesoscopic superconductor–semiconductor devices is crucial for their application in quantum technologies. Owing to their poor thermal conductivity, heating effects can drive superconducting-to-normal transitions even at low bias, observed as sharp conductance dips through the loss of Andreev excess currents. Tracking such dips across magnetic field, cryostat temperature, and applied microwave power allows us to uncover cooling bottlenecks in different parts of a device. By applying this “Joule spectroscopy” technique, we analyze heat dissipation in devices based on InAs-Al nanowires and reveal that cooling of superconducting islands is limited by the rather inefficient electron–phonon coupling, as opposed to grounded superconductors that primarily cool by quasiparticle diffusion. We show that powers as low as 50–150 pW are able to suppress superconductivity on the islands. Applied microwaves lead to similar heating effects but are affected by the interplay of the microwave frequency and the effective electron–phonon relaxation time.

Hybrid superconductor–semiconductor nanostructures have attracted great attention in the past decade as a platform for the development of novel quantum devices,^1,2^ with focus on both topological Majorana fermions^3−5^ and the study of conventional Andreev bound states,^6−9^ among others. Additionally, of great interest are mesoscopic superconducting islands, comprised of tunnel-coupled isolated regions with finite charging energy, which have been used to define charge qubits^10,11^ and to detect topological transitions^12−14^ and are promising for building artificial Kitaev chains.^15−17^ Common for these applications is a sensitivity to heightened temperature and nonequilibrium distributions of quasiparticles, which could introduce both quasiparticle (QP) poisoning and qubit decoherence.^18−25^ The dynamics of such out-of-equilibrium QPs in superconductors, also referred to as “hot-electrons”,^26−29^ are also responsible for the observed microcooling in SIN and SINIS devices,^30−34^ interesting for the pursuit of nanoscale thermodynamic elements beyond their detrimental effects in quantum computation, and likely lie at the origin of the suppression of superconductivity by electrostatic gating in metallic films.^25,35,36^

Despite great advances in both the fabrication and characterization of hybrid superconductor–semiconductor devices, the associated heating and relaxation mechanisms and their dependence on device geometry are still not well understood. Indeed, depending on the device and its environment, cooling can occur via different mechanisms, such as excited quasiparticles, limited at low temperatures by the superconducting gap, coupling to phonons, which is strongly suppressed by the weak electron–phonon coupling at low temperatures, or emission of photons.^37,38^ Here, we study such effects for the case of hybrid nanowire devices with superconducting islands, which, due to their electric isolation, also display more limited cooling channels than in open hybrid systems and, therefore, could potentially be more susceptible to heating.

While charge transport is rather straightforwardly measured through the application of voltage or current biases, heat transport is more subtle and requires both a well-controlled heat source and effective thermometers.^39,40^ By applying a DC current across a device, Joule power is deposited at resistive elements and, at given power thresholds, can drive superconducting parts to the normal state, when the local temperature reaches the critical superconducting temperature, *T* = *T*_*c*_. In devices with highly transmitting junctions, such superconductor-to-normal transitions can be observed as a rapid drop of the superconducting excess current, measurable via lock-in techniques.^41,42^ This, in total, yields both an electrically operated heat source and a single-shot thermometer at *T* = *T*_*c*_, which can be employed to investigate heating effects, a technique dubbed “Joule spectroscopy”.^43^ Furthermore, as *T*_*c*_ in full-shell nanowires is highly tunable through the Little–Parks effect,^44,45^ this single-shot measurement can be extended to a wider range of temperatures and powers. By applying this technique, we reveal the relevant heat dissipation mechanisms operating in grounded and floating superconductors in hybrid superconductor–semiconductor nanowire devices.

We focus on devices based on full-shell InAs-Al nanowires incorporating a mesoscopic superconducting island and present data corresponding to two devices in the main text. The first one, referred to as device A, was fabricated by wet etching two segments of approximately 200 nm of the Al shell, thus defining an island of length *L* ≈ 0.85 μm and two superconducting full-shell InAs-Al leads. Figure 1a displays a scanning electron micrograph of a device lithographically similar to device A (see Supporting Information for more details). Side gates *V*_*g*,*L*_ and *V*_*g*,*R*_ tune independently the transparency of each nanowire junction, and the plunger gate, *V*_*g*,*P*_, was fixed at 5 V for all measurements. As Joule spectroscopy relies on detecting changes in the excess current, we have carried out measurements in the open regime to maximize *I*_*exs*_. Specifically, we fix *V*_*g*,*L*_ = *V*_*g*,*R*_ = 5 V, obtaining a normal conductance *G*_*N*_ ≈ 4.5 × 2*e*^2^/*h* and *I*_*exs*_ ≈ 95 nA. We note that, in this regime, charging effects are also suppressed, and for this reason they are neglected in the remaining of our analysis. Finally, the application of an external magnetic field, *B*, which has a small angle of ≈2° with respect to the nanowire in device A, provides a powerful knob in our study. Indeed, the modulation of the superconducting critical temperatures, *T*_*c*_, of the island and of the leads by the Little–Parks effect^44,45^ will allow us to distinguish between different mechanisms for heat dissipation in our device.

**Figure 1:**
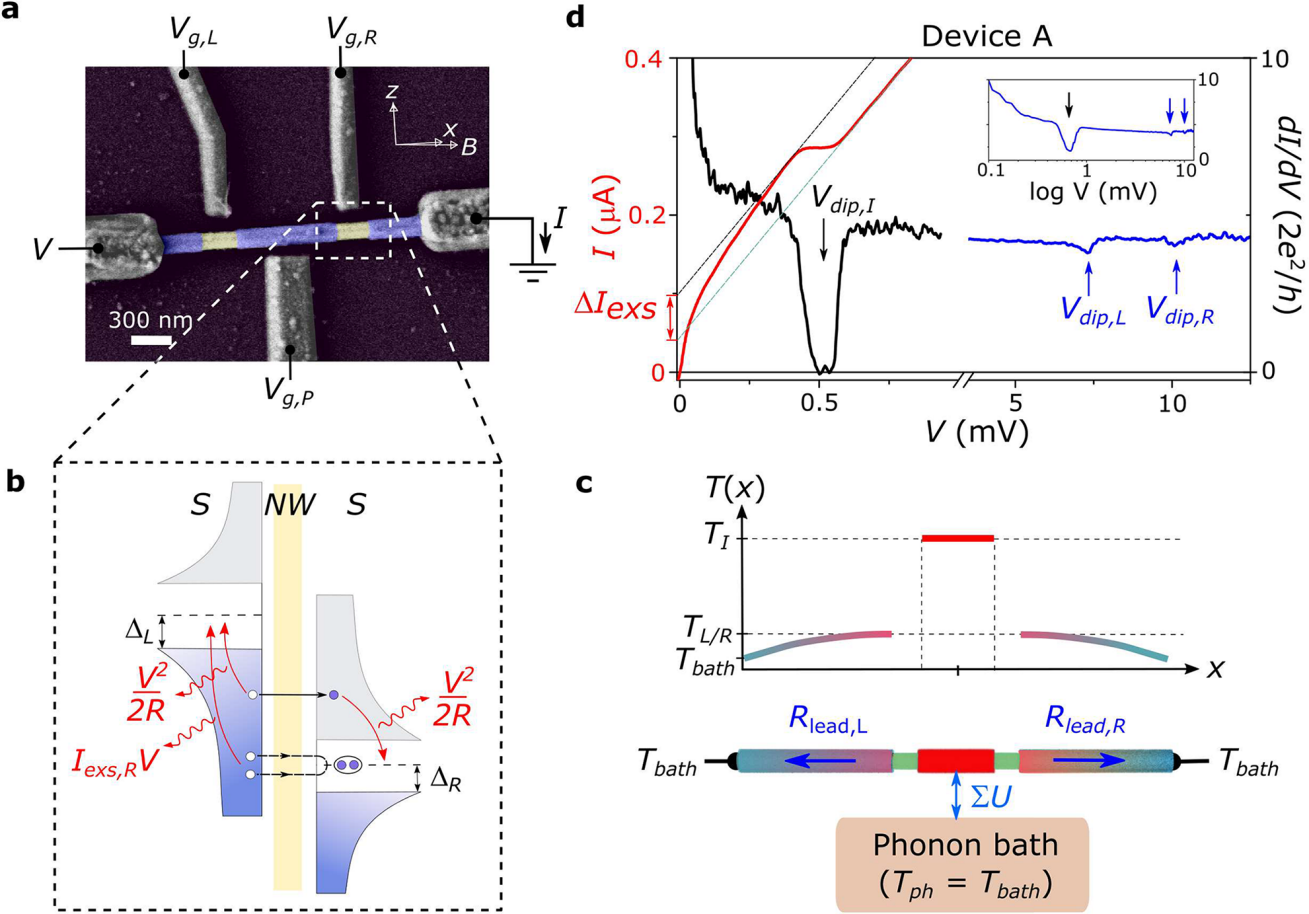
Joule heating in mesoscopic superconducting islands. (a) False-color electron micrograph of a device similar to device A. A superconducting island and two leads (purple) are defined in a full-shell InAs-Al nanowire. Side gates *V*_*g*,*L*_, *V*_*g*,*P*_, and *V*_*g*,*R*_ are fixed at 5 V for the measurements. The external magnetic field, *B*, is applied with a small angle with respect to the NW axis, *x*. (b) Scheme of different contributions to the Joule power deposited by the relaxation of hot quasiparticles in an *S*–*S* junction. The power deposited by single-electron processes, *V*^2^/*R*, is distributed evenly between left and right leads. By contrast, that associated with Andreev reflections, *I*_*exs*_*V*, is limited to only one side of the junction. (c) Schematics of the temperature distribution along the device due to Joule heating. *T*_*bath*_ is the cryostat temperature, whereas *T*_*j*_ (with *j* = *I*, *L*, *R*) are the temperatures at the island and on the left and right leads. Lower panel, thermal model with main cooling mechanisms: quasiparticle diffusion for the leads (governed by *R*_*lead*,*i*_) and electron–phonon coupling for the island (parametrized by Σ*U*). (c) *I*(*V*) (red line) and d*I*/d*V* (*V*) (black and blue lines) curves taken at *B* = 0. Three d*I*/d*V* dips are observed at *V*_*dip*,*I*_ ≈ 0.5 mV, *V*_*dip*,*L*_ ≈ 7.5 mV, and *V*_*dip*,*R*_ ≈ 10 mV, as underscored by arrows. They signal the transition of the superconductors to the normal state. A suppression of the excess current, Δ*I*_*exs*_, is observed in the *I*(*V*) curve when the island turns normal. Inset: d*I*/d*V* (*V*) measurement plotted against voltage in log scale. The black (blue) d*I*/d*V* curves were taken with d*V* = 5 μV (200 μV) to better resolve the low- (high-) bias dips (see Methods for more detail).

Let us start by discussing the impact of Joule heating on the temperature of hybrid superconductor–semiconductor devices. At the core of this problem lies the thermal balance between the amount of heat dissipated by the Joule effect and the existing cooling power in the device. Importantly, both heating and cooling powers may be distributed rather unevenly, as is the case for devices with superconducting islands. It is therefore useful to analyze the thermal balance at each of the superconductors separately:
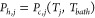
1where *P*_*h*,*j*_ and *P*_*c*,*j*_(*T*_*j*_, *T*_*bath*_), respectively, refer to the Joule power deposited on and the cooling power available at superconductor *j* (with *j* = *I*, *L*, *R* corresponding to the island, left lead, and right lead). Note that the latter depends on both the electronic temperature of the superconductor, *T*_*j*_, and the bath temperature, *T*_*bath*_, which we take to be equal to the cryostat temperature.

The heating considered here arises from the relaxation of hot quasiparticles generated in the superconductors by the flow of dissipative electrical currents (Figure 1b). For the sake of simplicity, we first discuss this effect for the case of a highly transmitting *S*–*S* junction. For *V* > (Δ_*L*_ + Δ_*R*_)/*e*, transport is well described by *I* = *V*/*R* + *I*_*exs*,*L*_ + *I*_*exs*,*R*_, where Δ_*L*_ and Δ_*R*_ are the superconducting gaps of the left and right leads and *R* is the normal resistance. *I*_*exs*,*L*_ (*I*_*exs*,*R*_) refers to the excess current stemming from Andreev reflections on the left (right) lead. As a result, the dissipated Joule power will have contributions from both ohmic and excess current terms above. Interestingly, while the former is distributed evenly between left and right leads, the excess current contributions are not. This owes to the fact that the Andreev reflections underlying *I*_*exs*_ generate quasiparticles only on the opposite side of the junction, as depicted in Figure 1b. Hence, the Joule power deposited on the left (right) lead is given by *P*_*h*,*L*_ = *V*^2^/2*R* + *I*_*exs*,*R*_*V* (*L* ↔ *R*). By extending the same logic to the geometry of device A (see Supporting Information for more details), we get
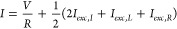
2
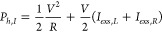
3
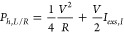
4where we assumed, for simplicity, that the resistances of the two nanowire junctions are equal, *R*_*L*_ = *R*_*R*_ = *R*/2, leading to approximately equal voltage drops across each junction, *V*/2. Note that the relations above satisfy the detailed balance, *IV* = *P*_*h*,*I*_ + *P*_*h*,*L*_ + *P*_*h*,*R*_, and demonstrate that the Joule power deposited on the three regions can be different.

At thermal equilibrium, the power deposited on superconductor *j* has to match *P*_*c*,*j*_(*T*_*j*_, *T*_*bath*_), the strength of which increases with *T*_*j*_. This means that if at a given instant *P*_*h*,*j*_ > *P*_*c*,*j*_(*T*_*j*_, *T*_*bath*_), then *T*_*j*_ rises to restore the balance in eq 1. In this way, upon increasing the Joule power, an inhomogeneous temperature distribution builds up in the device (Figure 1c), with hotter zones forming in regions with less efficient heat dissipation and/or a higher Joule power deposition. Clearly, for high enough Joule powers, the temperature can reach the superconducting critical temperature at a given superconductor, i.e., *T*_*j*_ = *T*_*c*,*j*_, driving its transition to the normal state. This occurs at a critical voltage, *V*_*dip*,*j*_, and leads to the full suppression of Andreev processes and *I*_*exs*,*j*_ = 0. Such a drop in current results in a sharp dip in differential conductance, d*I*/d*V*, at *V*_*dip*,*j*_, which is measured in the experiment. We detect three such d*I*/d*V* dips for device A (Figure 1d): one at relatively low bias (*V*_*dips*,*I*_ ≈ 0.5 mV), which we ascribe to the superconducting island, as it is more thermally isolated, and two appearing at higher bias (*V*_*dip*,*L*_ ≈ 7.5 mV and *V*_*dip*,*R*_ ≈ 10 mV), attributed to the left and right leads. A partial suppression of the excess current, Δ*I*_*exs*_, which correlates with the low-bias dip, is clearly identified in the *I*(*V*) curve in red, with *I*_*exs*_ finally going to zero for *V* ≳ 10 mV (not shown).

By applying eqs 3 and 4, we estimate the Joule powers required for driving the island and the leads of device A to the normal state, *P*_*dip*,*I*_ ≈ 60 pW, *P*_*dip*,*L*_ ≈ 5 nW, and *P*_*dip*,*R*_ ≈ 9 nW, respectively. Such a difference of around 2 orders of magnitude already suggests that the underlying heat dissipation mechanisms must be different. Following previous works,^42,43^ we ascribe quasiparticle diffusion as the dominant mechanism for the superconducting leads. Solving the corresponding heat diffusion equation yields the cooling power at *T*_*c*_ for the leads,
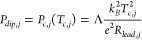
5with *R*_*lead*,*j*_ being the normal resistance of the leads and Λ = 2.112.^43^ By contrast, owing to the presence of the nanowire junctions, the superconducting island cannot cool down efficiently via quasiparticles. Instead, we assume that the heat dissipation predominantly occurs by electron–phonon coupling,
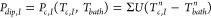
6where Σ is a material-dependent parameter, *U* is the volume of the island, and *n* is expected to range from 4 to 6, according to theory.^26,47−52^ Note that, due to their limited impact, other possible heating and cooling terms are neglected at this moment.

To test our assumptions regarding the dominant heat dissipation mechanisms for the superconducting island and leads, we study the dependence of the three d*I*/d*V* dips as a function of a magnetic field applied nearly parallel to the nanowire axis, as shown in Figure 2. Owing to the Little–Parks effect, the magnetic field gives rise to *T*_*c*,*j*_(*B*) oscillations, which translate into characteristic *V*_*dip*,*j*_(*B*) dependences for the distinct dissipation mechanisms. For example, quasiparticle diffusion yields *V*_*dip*,*j*_ directly proportional to *T*_*c*,*j*_, as concluded from eqs 4 and 5. We thus fit the high-bias dips in Figure 2a to *V*_*dip*,*L*/*R*_(*B*) ∝ *T*_*c*,*L*/*R*_(*B*) using Abrikosov–Gorkov (AG) theory^43,53^ (see Supporting Information for details of the fitting). The good observed agreement reinforces our interpretation of the dominant cooling mechanism for the leads at *T*_*c*_. Note that the fact that the two high-bias dips are not identical is due to asymmetries in the device, e.g., in *R*_*lead*,*j*_ or in *R*_*L*_, *R*_*R*_. The *B*-field behavior of the low-bias dip, on the other hand, does not follow the same dependence, as evidenced by the green dashed line in Figure 2b. Instead, we fit the experimental data to , which is obtained from eqs 3 and 6 by neglecting the excess current terms in *P*_*h*,*I*_ (which correspond to approximately 25% of total *P*_*h*,*I*_ in device A and may explain the lower *V*_*dip*,*I*_ observed in lobe 0 compared to lobe 1 in Figure 2b; see Supporting Information). A good agreement is achieved for *n* ∼ 5–6 (the red dashed line shows a fit for *n* = 6), with *T*_*c*,*I*_(*B*) obtained from AG theory and *T*_*bath*_ = 0, thus supporting our assumption of electron–phonon coupling as the main cooling mechanism in mesoscopic islands (see Supporting Information for fits with different values of *n*).

**Figure 2:**
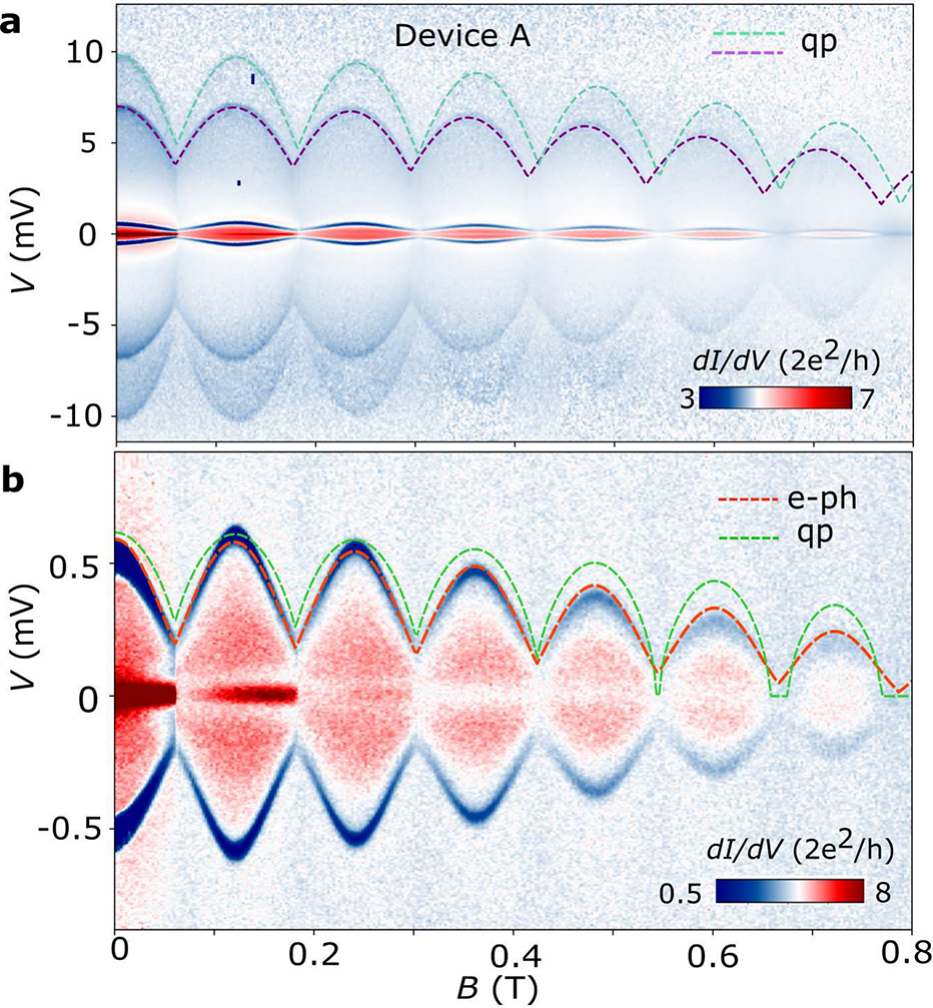
Heat dissipation mechanisms revealed by Joule spectroscopy. (a) d*I*/d*V* (*V*) as a function of magnetic field for device A, taken at a cryostat temperature of 20 mK. Owing to the Little–Parks effect, the positions of the three d*I*/d*V* dips oscillate with applied *B*. The dependence of the higher bias dips, *V*_*dip*,*L*/*R*_(*B*), which relate to the superconductor-to-normal transitions of the two full-shell InAs-Al leads, is directly proportional to *T*_*c*_(*B*), as underscored by the fits to the Abrikosov–Gork’ov theory (green and purple dashed lines, labeled as qp, referring to quasiparticle diffusion). By contrast, the magnetic field dependence of the lower bias dip, (*V*_*dip*,*I*_), in (b), is clearly distinct (the green dashed line is an attempt to fit the experimental data to *V*_*dip*,*I*_(*B*) ∝ *T*_*c*_(*B*)). A good agreement with the experimental data is obtained for  (red dashed line, labeled as e-ph, referring to electron–phonon coupling).

To further scrutinize heat dissipation in the islands, we have studied a second device (B), whose geometry incorporates two main modifications. First, we employ normal metal leads, which simplifies our analysis of the superconductor-to-normal transition of the island as the excess current terms in eq 3 disappear, and consequently *P*_*I*_ = (*R*_*L*_ + *R*_*R*_)*I*^2^/2 does not depend on the *R*_*L*_, *R*_*R*_ asymmetry. The device is fabricated by etching the Al shell at the ends of the nanowire and evaporating the Cr/Au leads, thus defining an *L* ≈ 1.4 μm-long island (see schematics in the inset of Figure 3b). Second, the device is fabricated with a thinner InAs-Al nanowire, which enters the destructive regime of the Little–Parks effect. This is useful as the differences between the quasiparticle diffusion and electron–phonon coupling dependences for *V*_*dip*,*I*_(*B*) grow with shrinking *T*_*c*,*I*_ as already observed for device A. To maximize the excess current, measurements were taken again in the open regime (where *V*_*g*_ = 10 V is the voltage applied to the global back gate, *G*_*N*_ ≈ 1.6 × 2*e*^2^/*h*, *I*_*exs*_ ≈ 25 nA). As device B contains only one superconducting element, a single d*I*/d*V* dip is observed, consistent with the full suppression of *I*_*exs*_ when the island turns normal. Figure 3 displays *V*_*dip*,*I*_(*B*) measurements taken at different cryostat temperatures, *T*_*bath*_ = 250, 600, 900, and 1200 mK. Note that here *V*_*dip*,*I*_ ≈ 1.5 mV and *P*_*dip*,*I*_ ≈ 150 pW at *B* = 0 and *T*_*bath*_ = 250 mK. We fit the experimental data in Figure 3a to *V*_*dip*,*I*_(*B*) ∝ *T*_*c*,*I*_(*B*) (green dashed line) and to  (red dashed line), where  is a fitting parameter, *T*_*bath*_ = 250 mK, and *T*_*c*,*I*_(*B*) is obtained by AG theory. A good agreement is obtained with the latter, from which we estimate Σ ≈ 4.3 × 10^9^ W/m^3^ K^6^ by taking *R* = 8.1 kΩ and *U* ≈ 4 × 10^–21^ m^3^ as the volume of the Al shell in the island. The estimated Σ is in good agreement with previous works.^50,54^ In the other panels, we plot *V*_*dip*,*I*_(*B*) calculated using β extracted above and the corresponding cryostat temperature as *T*_*bath*_ (dashed lines). The good correspondence for all measurements strongly supports our interpretation of electron–phonon coupling as the main cooling mechanism in islands at *T*_*c*_. Note that slight discrepancies in the measured and calculated *V*_*dip*,*I*_ appear for higher *T*_*bath*_, most clearly seen for the measurement taken at 1.2 K. This could be related, for example, to the impact of other weaker cooling/heating mechanisms neglected in our analysis and/or to temperature-dependent electron–phonon coupling parameters, e.g., Σ and *n*.^52^

**Figure 3:**
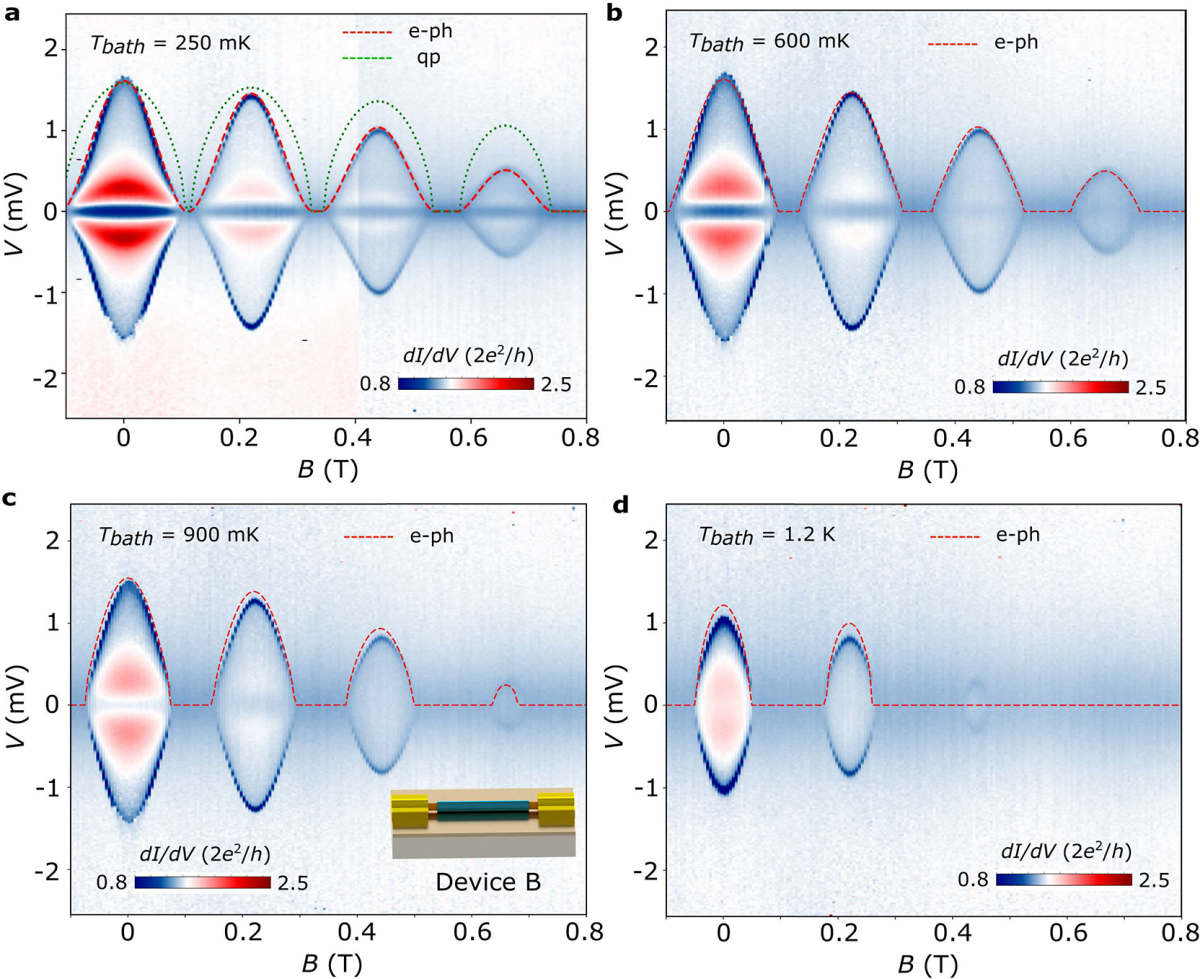
Electron–phonon coupling as the dominant heat dissipation mechanism in superconducting islands. d*I*/d*V* (*V*) as a function of the external magnetic field for device B at different cryostat temperatures: (a) 250 mK, (b) 600 mK, (c) 900 mK, and (d) 1.2 K. In this data set, a single d*I*/d*V* dip related to the island is observed, as the leads in this device are normal (Cr/Au, see inset in panel b). For the measurement taken at the lowest temperature (panel a), we plot the best fits to *V*_*dip*,*I*_ ∝ *T*_*c*_ (green dashed line, labeled as qp) and  (red dashed line, labeled as e-ph), using AG theory. *T*_*bath*_ is taken as the cryostat temperature. For the other panels, we plot *V*_*dip*,*I*_(*B*) calculated using the same fit parameters extracted from (a) and the corresponding *T*_*bath*_.

Finally, we address heating effects stemming from microwave radiation, which in our experiment is applied to an antenna located a few millimeters away from device A. By sweeping the frequency of the signal, we detect resonances at which a stronger effect on the dips is observed (see Supporting Information). In Figure 4a, we plot the impact of the AC signal amplitude, *V*_*rms*_, at one of such resonances (*f* = 3.492 GHz) on the superconductor-to-normal transitions of the islands and leads. All three dips move to lower voltages with increasing *V*_*rms*_, signaling that a lower DC Joule power is required for turning the superconductors normal, due to AC heating. To model the observed behavior we assume that the microwave is absorbed by circuit elements converting it into an effective AC voltage source, yielding a total bias *V*_*tot*_ = *V* + *V*_*AC*_ sin(2*πft*). For frequencies much faster than the thermalization rate, 1/τ_*th*_, a net Joule power, nominally given by *V*^2^/*R* + *V*_*AC*_^2^/2*R* when neglecting heating from excess current, is dissipated by the AC signal even though its voltage averages to zero over time. We simulate this using eqs 2–4, assuming cooling by quasiparticle diffusion in leads and by phonons in the island with parameters from DC fittings and using a smoothed step function to simulate the onset of excess current, and last, by using the average power of an AC cycle to self-consistently solve for the temperatures, *T*_*L*_, *T*_*R*_, and *T*_*I*_. The results of these calculations are shown in Figure 4b. The simulation captures very well the main qualitative features of the experimental data, including a splitting of the enhanced conductance at low bias following *V* = *V*_*AC*_ due to the onset of excess current at *V*_*tot*_ ≈ 0 being included in the conductance average for *f* ≫ 1/τ_*th*_. In Figure 4c, we evaluate the effect of the AC signal frequency on the low-bias dip. We notice a subtle yet important difference, namely, the splitting of the finite bias d*I*/d*V* dip for increasing *V*_*rms*_ in the lower frequency (*f* = 1 MHz) measurement. Indeed, for frequencies slower or comparable to thermalization, we would expect *T*_*I*_ to oscillate with the AC signal as the system seeks thermal equilibrium at each point of the AC cycle. In the limit of *f* ≪ 1/τ_*th*_, this would amount to a convolution of the DC result with , resulting in a dip splitting of *V* = *V*_*dip*,*I*_ ± |*V*_*AC*_|, consistent with our observations.

**Figure 4:**
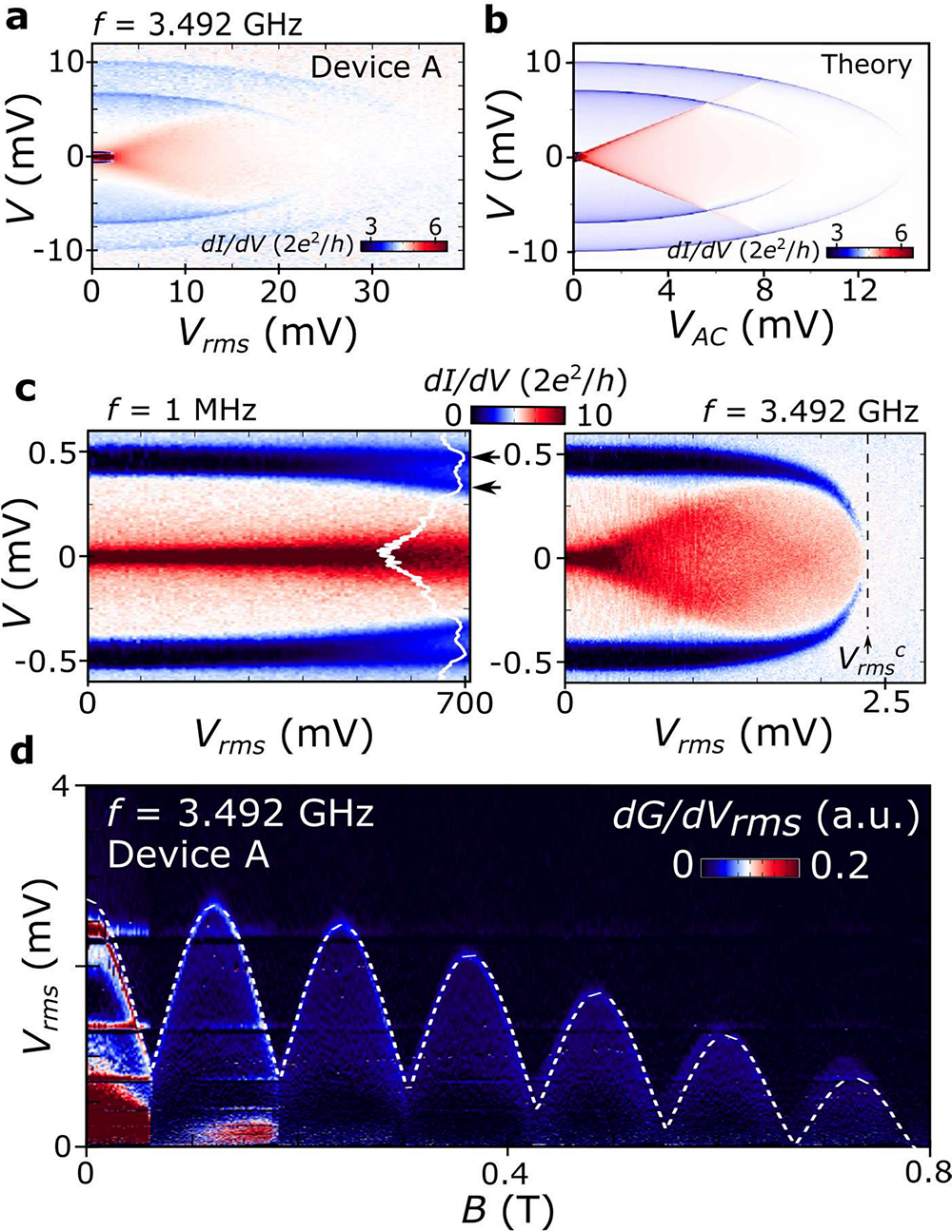
Heating effects with high-frequency AC signals. (a) d*I*/d*V* (*V*) of device A as a function of the nominal rms amplitude, *V*_*rms*_, of a *f* = 3.492 GHz AC signal applied to a nearby antenna (see Methods for more detail). (b) Theoretical simulation of d*I*/d*V* (*V*) as a function of the amplitude of an AC voltage signal, *V*_*AC*_, superimposed to the DC bias, *V*. (c) Impact of the AC signal frequency on the heating of the island. d*I*/d*V* (*V*) as a function of *V*_*rms*_ for *f* = 1 MHz (left panel) and 3.492 GHz (right panel). For the lower frequency, a splitting of the low-bias d*I*/d*V* dip is observed, also shown in the line cut taken at *V*_*rms*_ = 700 mV (white line), as highlighted by the black arrows. (d) *B*-field dependence of the microwave-induced superconductor-to-normal transition of the island at zero DC voltage. To improve the visibility of the transition, we plot the numerical derivative of the zero-bias conductance, *G* = d*I*/d*V* (*V* = 0), with respect to *V*_*rms*_, i.e., d*G*/d*V*_*rms*_ (see main text and Supporting Information for more detail). The white dashed line represents a fit to γ^–1^*V*_*dip*,*I*_(*B*), where *V*_*dip*,*I*_(*B*) is extracted from Figure 2b, and the AC coupling factor η is ≈0.2.

Assuming a harmonic solution to the heat-balance equation, we obtain the following expression for the thermal relaxation time,^55,56^
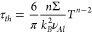
7with *n* being the power of eq 6, fitted to *n* = 6 in experiment, and ν_*Al*_ = 2.15 × 10^47^ J^–1^ m^–3^ the Fermi density of states of aluminum. For *T* = *T*_*c*_ = 1.35 K, appropriate for a dip, we find τ_*th*_ ≈ 10 ns, consistent with our measurement of fast and slow dynamics (see Supporting Information for further details).

For the measurement taken at higher *f*, a critical value of the AC signal amplitude, *V*_*rms*_^*c*^, can be identified for which the low-bias dip disappears after merging at *V* = 0. This critical amplitude represents the full suppression of the superconductivity of the island driven solely by AC heating. To study this in more detail, we take measurements of the differential conductance at zero bias, *G* = d*I*/d*V* (*V* = 0), as a function of *V*_*rms*_ and *B*. At *V*_*rms*_^*c*^, Josephson and Andreev processes are suppressed, leading to a drop in *G*. This critical point is better visualized by plotting d*G*/d*V*_*rms*_(*V*_*rms*_), as shown in Figure 4d, since this quantity approaches zero following the transition of the island. Strikingly, we observe that the *B*-field dependence of *V*_*rms*_^*c*^ is qualitatively identical to that of the DC voltage dip. This is underscored by the fit of the experimental data to η^–1^*V*_*dip*,*I*_(*B*), shown as a dashed line, which is carried out by employing *V*_*dip*,*I*_(*B*) from Figure 2b and yields an AC coupling factor, η ≈ 0.2, which would be 0.5 for a perfect AC voltage source in the *f* ≫ 1/τ_*th*_ regime. We therefore demonstrate that high-frequency AC signals give rise to Joule heating in a similar manner to that in the DC case.

We now briefly comment on neglected heating and cooling terms in our analysis. First, electron–phonon coupling is also present in the leads, but is estimated to be ∼100 times less efficient than quasiparticle diffusion from our *P*_*dip*,*j*_ measurements and is therefore negligible. Also, the temperature gradients across the junctions, given by *T*_*L*/*R*_ – *T*_*I*_, contribute to the cooling of the island and heating of the leads, as thermally activated quasiparticles cross the junction. This contribution is insubstantial for the leads, but can contribute to a cooling of about 10% of the phonon cooling for the island at *T*_*I*_ = *T*_*c*,*I*_, as discussed in the SI. For simplicity, we chose not to include this term, as its addition does not affect the quality of fits, and can therefore not be separated in experiment from phonon cooling, and would simply contribute to a slight rescaling of measured Σ. (For more details see Supporting Information.) Future experiments will be directed at addressing these more subtle effects, e.g., by studying devices with suspended nanowires.

Our measurements show that the temperature of superconductors in hybrid devices can increase significantly with the Joule power deposited from DC and/or microwave signals. Importantly, we observe that superconducting islands display a much stronger susceptibility toward heating, as their dissipation is limited by the weak electron–phonon coupling. Altogether, our observations suggest that nonequilibrium conditions and/or AC signals (including noise) can substantially impact the response of hybrid superconductor–semiconductor devices, especially in geometries with floating superconductors, by leading to increased local temperatures that can greatly exceed *T*_*bath*_. While these effects have been rather overlooked in relation to hybrid devices, they can have implications, for example, in geometries proposed for the realization of topological superconducting phases and related qubits.^12−15,57,58^ We note that while our experiment focuses on extreme conditions at which the superconducting elements of a device transit to the normal state, heating effects are even more pronounced for lower (AC/DC) applied powers, as both quasiparticle diffusion and electron–phonon coupling are suppressed with decreasing electron temperature. Revealing and quantifying heat dissipation mechanisms as we do in this work provide a first step toward their control in devices for quantum applications and for basic research on quantum thermodynamics.^40,59^

When finalizing the preparation of this manuscript, we became aware of this related work.^60^

## Supporting Information Available

Materials, methods (device fabrication, description of experimental setups, data processing, description of fits to Little–Parks oscillations), transport theory, supplementary data, Figures S1–S10 (temperature dependence of the high- and low-bias dips for device A; comparison between *V*- and *I*-bias measurements taken in the ^3^He insert cryogenic system; resonances in the coupling between microwave signals and device A; data of AC signal heating without numerical differentiation; gate dependence of high- and low-bias dips for device A; fits to *V*_*dip*,*j*_(*B*) for device A; fits to *V*_*dip*,*j*_(*B*) for device B; simulation of device A low- and high-bias dips; comparisons of lobe 0 and lobe 1; comparison of slow and fast AC) (PDF)

## References

[ref1] Prada E.; San-Jose P.; de Moor M. W. A.; Geresdi A.; Lee E. J. H.; Klinovaja J.; Loss D.; Nygård J.; Aguado R.; Kouwenhoven L. P. From Andreev to Majorana bound states in hybrid superconductor–semiconductor nanowires. Nat. Rev. Phys. 2020, 2, 575–594. 10.1038/s42254-020-0228-y.

[ref2] Aguado R. A perspective on semiconductor-based superconducting qubits. Appl. Phys. Lett. 2020, 117, 240501 10.1063/5.0024124.

[ref3] Sau J. D.; Lutchyn R. M.; Tewari S.; Das Sarma S. Generic New Platform for Topological Quantum Computation Using Semiconductor Heterostructures. Phys. Rev. Lett. 2010, 104, 040502 10.1103/PhysRevLett.104.040502. 20366693

[ref4] Lutchyn R. M.; Sau J. D.; Das Sarma S. Majorana Fermions and a Topological Phase Transition in Semiconductor-Superconductor Heterostructures. Phys. Rev. Lett. 2010, 105, 077001 10.1103/PhysRevLett.105.077001. 20868069

[ref5] Oreg Y.; Refael G.; von Oppen F. Helical Liquids and Majorana Bound States in Quantum Wires. Phys. Rev. Lett. 2010, 105, 177002 10.1103/PhysRevLett.105.177002. 21231073

[ref6] Tosi L.; Metzger C.; Goffman M. F.; Urbina C.; Pothier H.; Park S.; Yeyati A. L.; Nygård J.; Krogstrup P. Spin-Orbit Splitting of Andreev States Revealed by Microwave Spectroscopy. Phys. Rev. X 2019, 9, 011010 10.1103/PhysRevX.9.011010.

[ref7] Hays M.; Fatemi V.; Bouman D.; Cerrillo J.; Diamond S.; Serniak K.; Connolly T.; Krogstrup P.; Nygård J.; Yeyati A. L.; Geresdi A.; Devoret M. H. Coherent manipulation of an Andreev spin qubit. Science 2021, 373, 430–433. 10.1126/science.abf0345. 34437115

[ref8] Wesdorp J. J.; Grünhaupt L.; Vaartjes A.; Pita-Vidal M.; Bargerbos A.; Splitthoff L. J.; Krogstrup P.; van Heck B.; de Lange G. Dynamical Polarization of the Fermion Parity in a Nanowire Josephson Junction. Phys. Rev. Lett. 2023, 131, 117001 10.1103/PhysRevLett.131.117001. 37774257

[ref9] Pita-Vidal M.; Bargerbos A.; Žitko R.; Splitthoff L. J.; Grünhaupt L.; Wesdorp J. J.; Liu Y.; Kouwenhoven L. P.; Aguado R.; van Heck B.; Kou A.; Andersen C. K. Direct manipulation of a superconducting spin qubit strongly coupled to a transmon qubit. Nat. Phys. 2023, 19, 1110–1115. 10.1038/s41567-023-02071-x.

[ref10] Bouchiat V.; Vion D.; Joyez P.; Esteve D.; Devoret M. H. Quantum coherence with a single Cooper pair. Phys. Scr. 1998, 1998, 165 10.1238/Physica.Topical.076a00165.

[ref11] Makhlin Y.; Schön G.; Shnirman A. Quantum-state engineering with Josephson-junction devices. Rev. Mod. Phys. 2001, 73, 357–400. 10.1103/RevModPhys.73.357.

[ref12] Albrecht S. M.; Higginbotham A. P.; Madsen M.; Kuemmeth F.; Jespersen T. S.; Nygård J.; Krogstrup P.; Marcus C. M. Exponential protection of zero modes in Majorana islands. Nature 2016, 531, 206–209. 10.1038/nature17162. 26961654

[ref13] Sherman D.; Yodh J. S.; Albrecht S. M.; Nygård J.; Krogstrup P.; Marcus C. M. Normal, superconducting and topological regimes of hybrid double quantum dots. Nat. Nanotechnol. 2017, 12, 212–217. 10.1038/nnano.2016.227. 27842064

[ref14] Valentini M.; Borovkov M.; Prada E.; Martí-Sánchez S.; Botifoll M.; Hofmann A.; Arbiol J.; Aguado R.; San-Jose P.; Katsaros G. Majorana-like Coulomb spectroscopy in the absence of zero-bias peaks. Nature 2022, 612, 442–447. 10.1038/s41586-022-05382-w. 36517713

[ref15] Sau J. D.; Sarma S. D. Realizing a robust practical Majorana chain in a quantum-dot-superconductor linear array. Nat. Commun. 2012, 3, 1–6. 10.1038/ncomms1966. 22805571

[ref16] Dvir T.; et al. Realization of a minimal Kitaev chain in coupled quantum dots. Nature 2023, 614, 445–450. 10.1038/s41586-022-05585-1. 36792741

[ref17] Estrada Saldaña J. C.; Vekris A.; Pavešić L.; Krogstrup P.; Žitko R.; Grove-Rasmussen K.; Nygård J. Excitations in a superconducting Coulombic energy gap. Nat. Commun. 2022, 13, 1–8. 10.1038/s41467-022-29634-5. 35473891 PMC9042936

[ref18] Serniak K.; Hays M.; De Lange G.; Diamond S.; Shankar S.; Burkhart L.; Frunzio L.; Houzet M.; Devoret M. Hot Nonequilibrium Quasiparticles in Transmon Qubits. Phys. Rev. Lett. 2018, 121, 157701 10.1103/PhysRevLett.121.157701. 30362798

[ref19] Bargerbos A.; Splitthoff L. J.; Pita-Vidal M.; Wesdorp J. J.; Liu Y.; Krogstrup P.; Kouwenhoven L. P.; Andersen C. K.; Grünhaupt L. Mitigation of Quasiparticle Loss in Superconducting Qubits by Phonon Scattering. Phys. Rev. Appl. 2023, 19, 024014 10.1103/PhysRevApplied.19.024014.

[ref20] Karzig T.; Cole W. S.; Pikulin D. I. Quasiparticle Poisoning of Majorana Qubits. Phys. Rev. Lett. 2021, 126, 057702 10.1103/PhysRevLett.126.057702. 33605758

[ref21] Catelani G.; Basko D. M. Non-equilibrium quasiparticles in superconducting circuits: photons vs. phonons. SciPost Phys. 2019, 6, 013 10.21468/SciPostPhys.6.1.013.

[ref22] Dong Y.; Li Y.; Zheng W.; Zhang Y.; Ma Z.; Tan X.; Yu Y. Measurement of Quasiparticle Diffusion in a Superconducting Transmon Qubit. Appl. Sci. 2022, 12, 8461 10.3390/app12178461.

[ref23] Le Calvez K.; Veyrat L.; Gay F.; Plaindoux P.; Winkelmann C. B.; Courtois H.; Sacépé B. Joule overheating poisons the fractional ac Josephson effect in topological Josephson junctions. Commun. Phys. 2019, 2, 4 10.1038/s42005-018-0100-x.

[ref24] De Cecco A.; Le Calvez K.; Sacépé B.; Winkelmann C. B.; Courtois H. Interplay between electron overheating and ac Josephson effect. Phys. Rev. B 2016, 93, 180505 10.1103/PhysRevB.93.180505.

[ref25] Alegria L. D.; Bo̷ttcher C. G. L.; Saydjari A. K.; Pierce A. T.; Lee S. H.; Harvey S. P.; Vool U.; Yacoby A. High-energy quasiparticle injection into mesoscopic superconductors. Nat. Nanotechnol. 2021, 16, 404–408. 10.1038/s41565-020-00834-8. 33462428

[ref26] Wellstood F. C.; Urbina C.; Clarke J. Hot-electron effects in metals. Phys. Rev. B 1994, 49, 5942–5955. 10.1103/PhysRevB.49.5942. 10011570

[ref27] Pekola J. P.; Saira O.-P.; Maisi V. F.; Kemppinen A.; Möttönen M.; Pashkin Y. A.; Averin D. V. Single-electron current sources: Toward a refined definition of the ampere. Rev. Mod. Phys. 2013, 85, 1421–1472. 10.1103/RevModPhys.85.1421.

[ref28] Zgirski M.; Foltyn M.; Savin A.; Naumov A.; Norowski K. Heat Hunting in a Freezer: Direct Measurement of Quasiparticle Diffusion in Superconducting Nanowire. Phys. Rev. Appl. 2020, 14, 044024 10.1103/PhysRevApplied.14.044024.

[ref29] Pothier H.; Guéron S.; Birge N. O.; Esteve D.; Devoret M. H. Energy Distribution Function of Quasiparticles in Mesoscopic Wires. Phys. Rev. Lett. 1997, 79, 3490–3493. 10.1103/PhysRevLett.79.3490.

[ref30] Pannetier B.; Courtois H.; Rajauria S. Quasiparticle diffusion based heating in superconductor tunneling micro-coolers. Phys. Rev. B 2009, 80, 214521 10.1103/PhysRevB.80.214521.

[ref31] Pekola J. P.; Anghel D. V.; Suppula T. I.; Suoknuuti J. K.; Manninen A. J.; Manninen M. Trapping of quasiparticles of a nonequilibrium superconductor. Appl. Phys. Lett. 2000, 76, 2782–2784. 10.1063/1.126474.

[ref32] Courtois H.; Meschke M.; Peltonen J. T.; Pekola J. P. Origin of Hysteresis in a Proximity Josephson Junction. Phys. Rev. Lett. 2008, 101, 067002 10.1103/PhysRevLett.101.067002. 18764493

[ref33] Aref T.; Maisi V. F.; Gustafsson M. V.; Delsing P.; Pekola J. P. Andreev tunneling in charge pumping with SINIS turnstiles. EPL (Europhys. Lett.) 2011, 96, 37008 10.1209/0295-5075/96/37008.

[ref34] Knowles H. S.; Maisi V. F.; Pekola J. P. Probing quasiparticle excitations in a hybrid single electron transistor. Appl. Phys. Lett. 2012, 100, 262601 10.1063/1.4730407.

[ref35] De Simoni G.; Paolucci F.; Solinas P.; Strambini E.; Giazotto F. Metallic supercurrent field-effect transistor. Nat. Nanotechnol. 2018, 13, 802–805. 10.1038/s41565-018-0190-3. 29967460

[ref36] Elalaily T.; Berke M.; Kedves M.; Fülöp G.; Scherübl Z.; Kanne T.; Nygård J.; Makk P.; Csonka S. Signatures of Gate-Driven Out-of-Equilibrium Superconductivity in Ta/InAs Nanowires. ACS Nano 2023, 17, 5528–5535. 10.1021/acsnano.2c10877. 36912466 PMC10062030

[ref37] Schmidt D. R.; Schoelkopf R. J.; Cleland A. N. Photon-Mediated Thermal Relaxation of Electrons in Nanostructures. Phys. Rev. Lett. 2004, 93, 045901 10.1103/PhysRevLett.93.045901. 15323773

[ref38] Meschke M.; Guichard W.; Pekola J. P. Single-mode heat conduction by photons. Nature 2006, 444, 187–190. 10.1038/nature05276. 17093446

[ref39] Pekola J. P.; Karimi B. Colloquium: Quantum heat transport in condensed matter systems. Rev. Mod. Phys. 2021, 93, 041001 10.1103/RevModPhys.93.041001.

[ref40] Karimi B.; Pekola J. P. Noninvasive Thermometer Based on the Zero-Bias Anomaly of a Superconducting Junction for Ultrasensitive Calorimetry. Phys. Rev. Appl. 2018, 10, 054048 10.1103/PhysRevApplied.10.054048.

[ref41] Lee H.-J.; Choi J.-H.; Doh Y.-J. Above-gap Conductance Anomaly Studied in Superconductor-graphene-superconductor Josephson Junctions. J. Korean Phys. Soc. 2010, 57, 149–155. 10.3938/jkps.57.149.

[ref42] Tomi M.; Samatov M. R.; Vasenko A. S.; Laitinen A.; Hakonen P.; Golubev D. S. Joule heating effects in high transparency Josephson junctions. Phys. Rev. B 2021, 104, 134513 10.1103/PhysRevB.104.134513.

[ref43] Ibabe A.; Gómez M.; Steffensen G. O.; Kanne T.; Nygård J.; Yeyati A. L.; Lee E. J. H. Joule spectroscopy of hybrid superconductor–semiconductor nanodevices. Nat. Commun. 2023, 14, 2873 10.1038/s41467-023-38533-2. 37208316 PMC10199083

[ref44] Little W. A.; Parks R. D. Observation of Quantum Periodicity in the Transition Temperature of a Superconducting Cylinder. Phys. Rev. Lett. 1962, 9, 9–12. 10.1103/PhysRevLett.9.9.

[ref45] Vaitiekėnas S.; Winkler G. W.; van Heck B.; Karzig T.; Deng M.-T.; Flensberg K.; Glazman L. I.; Nayak C.; Krogstrup P.; Lutchyn R. M.; Marcus C. M. Flux-induced topological superconductivity in full-shell nanowires. Science 2020, 367, eaav3392 10.1126/science.aav3392. 32217701

[ref47] Reizer M. Y. Electron-phonon relaxation in pure metals and superconductors at very low temperatures. Phys. Rev. B 1989, 40, 5411–5416. 10.1103/PhysRevB.40.5411. 9992571

[ref48] Lemzyakov S. A.; Tarasov M. A.; Edelman V. S. Electron-Phonon Interaction in Aluminum SINIS. IEEE Trans. Appl. Supercond. 2022, 32, 1–4. 10.1109/TASC.2021.3139261.

[ref49] O’Neil G. C.; Lowell P. J.; Underwood J. M.; Ullom J. N. Measurement and modeling of a large-area normal-metal/insulator/superconductor refrigerator with improved cooling. Phys. Rev. B 2012, 85, 134504 10.1103/PhysRevB.85.134504.

[ref50] Subero D.; Maillet O.; Golubev D. S.; Thomas G.; Peltonen J. T.; Karimi B.; Marín-Suárez M.; Yeyati A. L.; Sánchez R.; Park S.; Pekola J. P. Bolometric detection of Josephson inductance in a highly resistive environment. Nat. Commun. 2023, 14, 1–8. 10.1038/s41467-023-43668-3. 38040683 PMC10692220

[ref51] Karvonen J. T.; Taskinen L. J.; Maasilta I. J. Observation of disorder-induced weakening of electron-phonon interaction in thin noble metal films. Phys. Rev. B 2005, 72, 012302 10.1103/PhysRevB.72.012302.

[ref52] Underwood J. M.; Lowell P. J.; O’Neil G. C.; Ullom J. N. Insensitivity of Sub-Kelvin Electron-Phonon Coupling to Substrate Properties. Phys. Rev. Lett. 2011, 107, 255504 10.1103/PhysRevLett.107.255504. 22243092

[ref53] Abrikosov A. A.; Gor’kov L. P. Contribution to the theory of superconducting alloys with paramagnetic impurities. Zh. Eksp. Teor. Fiz 1960, 39, 1781.

[ref54] Karvonen J. T.; Maasilta I. J. Observation of phonon dimensionality effects on electron energy relaxation. J. Phys.: Conf. Ser. 2007, 92, 012043 10.1088/1742-6596/92/1/012043. 17930684

[ref55] O’Neil G. C.; Lowell P. J.; Underwood J. M.; Ullom J. N. Measurement and modeling of a large-area normal-metal/insulator/superconductor refrigerator with improved cooling. Phys. Rev. B 2012, 85, 134504 10.1103/PhysRevB.85.134504.

[ref56] Viisanen K. L.; Pekola J. P. Anomalous electronic heat capacity of copper nanowires at sub-Kelvin temperatures. Phys. Rev. B 2018, 97, 115422 10.1103/PhysRevB.97.115422.

[ref57] Aasen D.; Hell M.; Mishmash R. V.; Higginbotham A.; Danon J.; Leijnse M.; Jespersen T. S.; Folk J. A.; Marcus C. M.; Flensberg K.; Alicea J. Milestones toward Majorana-based quantum computing. Phys. Rev. X 2016, 6, 031016 10.1103/PhysRevX.6.031016.

[ref58] Whiticar A. M.; Fornieri A.; O’Farrell E. C. T.; Drachmann A. C. C.; Wang T.; Thomas C.; Gronin S.; Kallaher R.; Gardner G. C.; Manfra M. J.; Marcus C. M.; Nichele F. Coherent transport through a Majorana island in an Aharonov–Bohm interferometer. Nat. Commun. 2020, 11, 3212 10.1038/s41467-020-16988-x. 32587242 PMC7316771

[ref59] Gümüş E.; Majidi D.; Nikolić D.; Raif P.; Karimi B.; Peltonen J. T.; Scheer E.; Pekola J. P.; Courtois H.; Belzig W.; Winkelmann C. B. Calorimetry of a phase slip in a Josephson junction. Nat. Phys. 2023, 19, 196–200. 10.1038/s41567-022-01844-0.

[ref60] Shpagina E. V.; Tikhonov E. S.; Ruhstorfer D.; Koblmüller G.; Khrapai V. S. Fate of the superconducting state in floating islands of hybrid nanowire devices. Phys. Rev. B 2024, 109, L140501 10.1103/PhysRevB.109.L140501.

