# Supplementary material for: Heat Dissipation Mechanisms in Hybrid Superconductor–Semiconductor Devices Revealed by Joule Spectroscopy
Source: Nano Lett. 2024 May 21;24(22):6488–95. doi: 10.1021/acs.nanolett.4c00574 (PMC11157656; doi:10.1021/acs.nanolett.4c00574)
Supplement: Supplementary file 1 — nl4c00574_si_001.pdf [file nl4c00574_si_001.pdf]

Supporting Information
Heat dissipation mechanisms in hybrid superconductor-semiconductor devices revealed by Joule spectroscopy

Ángel Ibabe,[1,3] Gorm O. Steffensen,[2,3] Ignacio Casal,[1,3] Mario Gómez,[1,3] Thomas Kanne,[5] Jesper Nygård,[5] Alfredo Levy Yeyati,[2,3,4] and Eduardo J. H. Lee[1,3,4,*]

[1]*Departamento de Física de la Materia Condensada, Universidad Autónoma de Madrid, E-28049 Madrid, Spain*

[2]*Departamento de Física Teórica de la Materia Condensada, Universidad Autónoma de Madrid, E-28049 Madrid, Spain*

[3]*Condensed Matter Physics Center (IFIMAC), Universidad Autónoma de Madrid, E-28049 Madrid, Spain*

[4]*Instituto Nicolás Cabrera, Universidad Autónoma de Madrid, E-28049 Madrid, Spain*

[5]*Center for Quantum Devices, Niels Bohr Institute, University of Copenhagen, DK-2100 Copenhagen, Denmark*

CONTENTS

S1. Methods S3
A. **Sample fabrication and measured samples** S3
B. **Measurements and setup** S3
C. **Data processing** S4

S2. Supplementary data figures S5

S3. Gate dependence of conductance dips S10

S4. Obtaining device parameters by fitting Little-Parks oscillations S12

S5. Transport theory S16
A. Cooling mechanisms S17
B. Discussion of device A lobe 0 discrepancy S20
C. Effects of Microwave S22

References S23

* eduardo.lee@uam.es

S1. METHODS

A. Sample fabrication and measured samples

The devices discussed in the main text are based on full-shell InAs-Al nanowires and incorporate a mesoscopic superconducting island. By fitting the Little-Parks oscillations of the devices, we estimate the diameter of the InAs nanowire core to be $\approx$ 150 nm ($\approx$ 110 nm) for device A (B), and the thickness of the Al shell to be $\approx$ 8 nm.

The nanowires were deterministically transferred using a micro-manipulator from the growth chip to silicon substrates for further fabrication. Specifically, intrinsic (degenerately-doped) Si substrates with 300 nm-thick SiO_2 dielectrics were employed for device A (B).

Standard e-beam lithography (EBL) was used to define masks for wet etching segments of the epitaxial shell. Oxygen plasma descumming at 100 W for 75 s was carried out before immersing the samples in AZ326 MIF developer (containing 2.38% tetramethylammonium hydroxide, TMAH) for 75 s at room temperature. For device A, two segments of $\approx$ 200 nm of the Al shell were etched, defining the superconducting island and two superconducting leads. By contrast, for device B, we removed completely the Al shell at the ends of the nanowire to form the island, and to allow the fabrication of normal metal leads at a subsequent process. Electrical contacts and side gates were fabricated by standard EBL techniques, followed by metallization by e-beam evaporation at pressures of $\sim 10^{-8}$ mbar. Ion milling is carried out prior to the evaporation to remove the native oxide of the Al shell (InAs nanowire) for device A (B). The evaporated electrical contacts consisted of superconducting Ti (2.5 nm)/Al (240 nm) for device A, and normal Cr (2.5 nm)/Au (180 nm) for device B. Note that, for device B, the Cr/Au leads are fabricated $\approx$ 200 nm away from the superconducting island, and that the doped Si substrate was used as a global back gate.

The main features discussed in this work have been observed in at least 4 devices similar to device A, and 2 similar to device B.

B. Measurements and setup

Our experiments were carried out in two different cryogenic systems: a dilution refrigerator with a base temperature of 20 mK, which was employed for measuring device A, and

a ^{3}He insert with a base temperature of 250 mK, employed in the measurement of device B. The cryostat temperatures were measured by ruthenium oxide thermometers attached to the ^{3}He pot and the mixing chamber of the above systems.

All data related to device A was obtained by performing two-terminal voltage-bias transport measurements using standard lock-in techniques, whereby a voltage, V, is applied with a low-amplitude, low-frequency ($f = 117$ Hz) AC excitation, dV, and the current, I, and differential conductance, dI/dV are measured. We have employed different amplitudes of the lock-in excitation depending on whether the measurement targeted resolving the low-bias dip only ($dV = 5\ \mu$V), or also the high-bias dips ($dV = 100$ - $200\ \mu$V), as the contrast of the latter is much weaker. (Note: the dV values listed above are the nominal ones, i.e., before subtracting the voltage drop on the cryogenic filters, as we explain below).

For device B, we have experimented with both two-terminal voltage-bias and current-bias measurements. We have observed that the experimental data obtained with these two different schemes are completely equivalent, e.g., when comparing measurements plotted: (i) as a function of the applied voltage-bias or of the voltage drop across the device as a result of an applied current-bias, or (ii) as a function of the current resulting from an applied voltage-bias or of the applied current-bias. In the main text, we have opted to display the two-terminal current-bias measurements, owing to a reduced electrical noise in this configuration for our ^{3}He insert setup, which translated into sharper dI/dV dips. For this reason, V in Fig. 3 refers to the total voltage drop across device B resulting from an applied current-bias, I. These measurements were taken with a lock-in excitation equal to $dI = 1$ nA at $f = 117$ Hz.

Microwave radiation from a signal generator (Rohde & Schwarz SMW200A) was applied to an on-chip antenna located a few millimeters from device A. The coupling between the devices and the impedance of the microwave antenna is not precisely characterized. Therefore, the given V_{rms} values in the main text are nominal values obtained as $V_{rms} \propto 10^{P/20}\sqrt{P_0}$ with $P_0 = 1$mW and P the nominal power in dBm units.

C. Data processing

The voltage drop on the total series resistance of our experimental setups, which is primarily due to cryogenic filters (2.5 kΩ per experimental line), has been subtracted for plotting

the data shown in Figs. 1-4.

S2. SUPPLEMENTARY DATA FIGURES

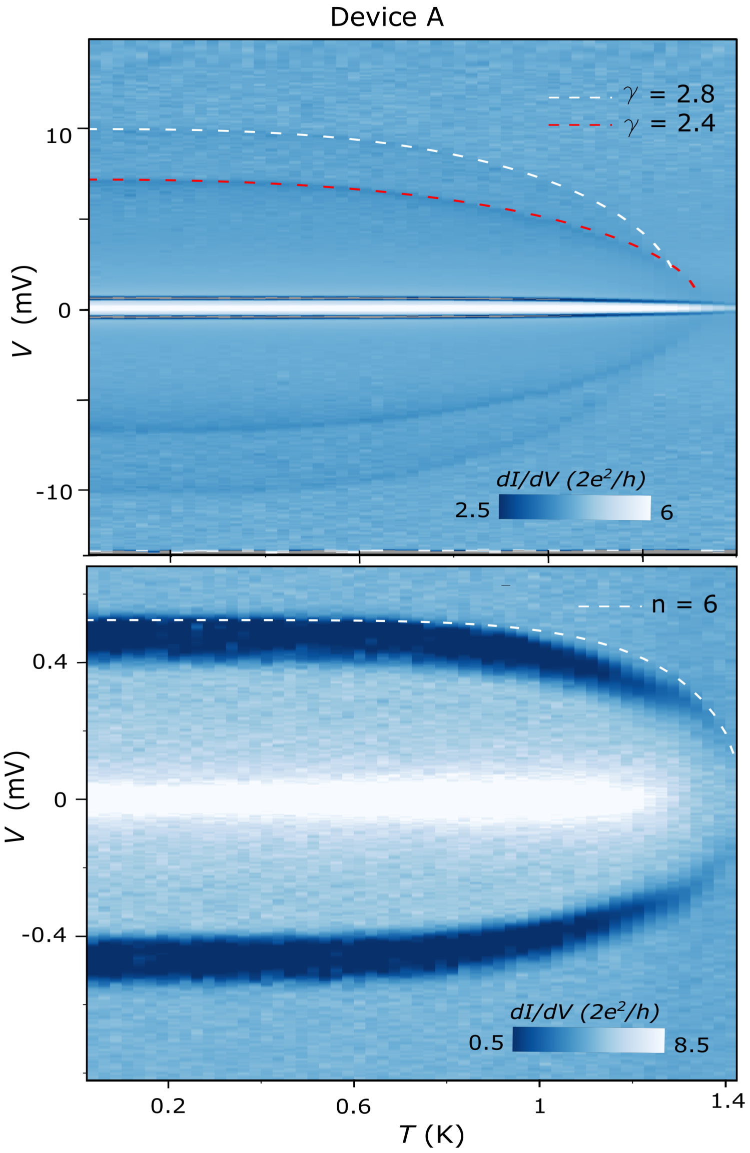

FIG. S1. **Temperature dependence of the high- and low-bias dips for device A.** Top panel, $dI/dV(V)$ measured as a function of cryostat temperature. The three dI/dV dips ($V_{dip,L} \approx 7.5$ mV, $V_{dip,R} \approx 10$ mV, and $V_{dip,I} \approx 0.5$ mV) move to lower voltages with increasing temperature and finally disappear at $T_{bath} = T_{c,j}$. Fits to $\gamma = 2.8$ and $\gamma = 2.4$ are obtained (see Section 4.A for discussion). A similar measurement (bottom), highlights the low-bias dip. A fit of this dip to Eq. (6) of the main text with $n = 6$ is plotted as a dashed white line. As discussed in the main text, slight deviations are observed between the experimental data and the fit as T approaches T_c. The lock-in excitation was fixed at 200 μV (5 μV) for the top (bottom) panel.

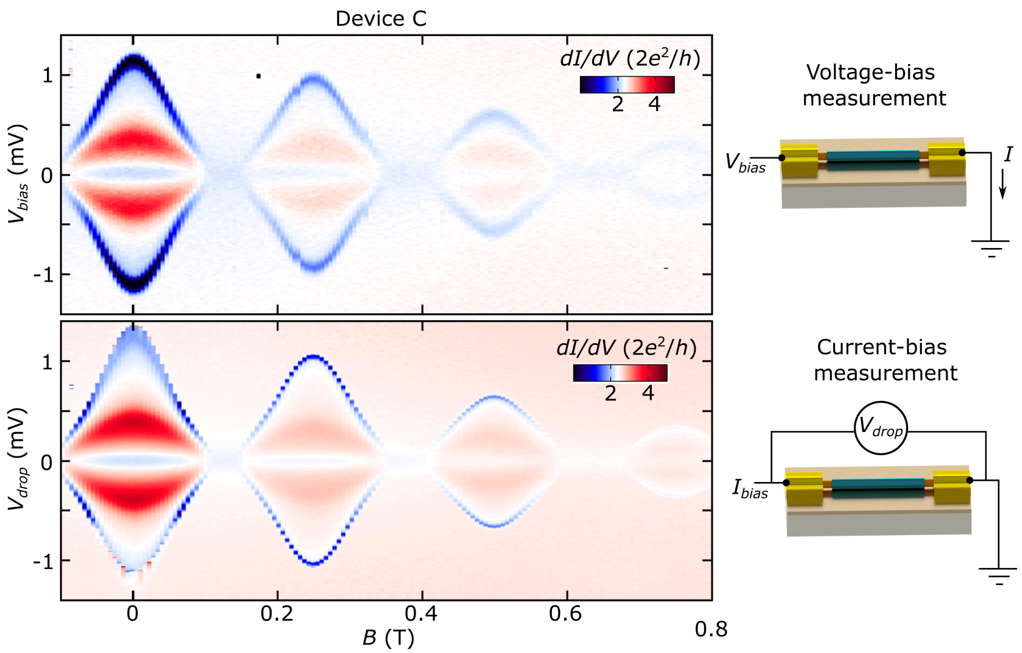

FIG. S2. **Comparison between voltage- and current-bias measurements taken in the ^{3}He insert cryogenic system.** B-field dependence of dI/dV plotted either as a function of the voltage-bias, V_{bias} (top) or the voltage-drop resulting from a current-bias, V_{drop} (bottom) for a third device C with the same geometry as device B in the main text. Both experimental datasets are equivalent but, owing to the reduced noise of the current-bias setup in this cryostat, a better resolution of the dI/dV dips is obtained, favoring the fits described in the main text. For this reason, we employed the current-bias setup for studying device B.

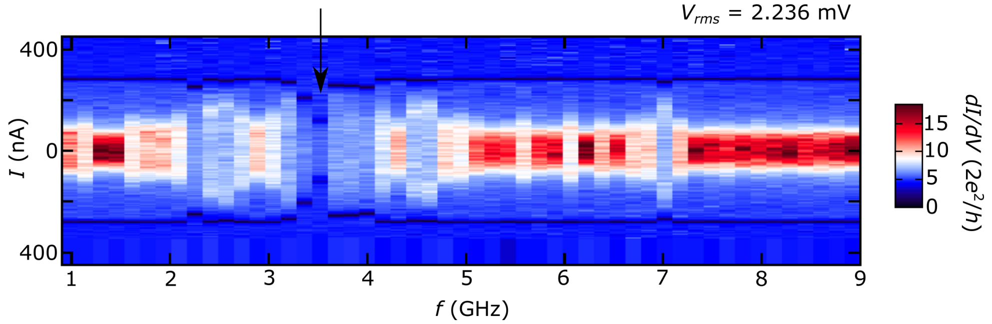

FIG. S3. **Resonances in the coupling between microwave signals and device A.** $dI/dV(I)$ measured as a function of the microwave signal frequency, f, applied to an antenna located a few millimeters away from the device at a nominal power $P = -40$ dBm ($V_{rms} \approx 2.236$ mV). The effect of the microwave signal on $V_{dip,I}$ depends strongly on the AC frequency, with resonances occurring for given values of f. The measurements shown in the main text were taken by fixing the frequency at one of such resonances ($f = 3.492$ GHz, highlighted by the arrow).

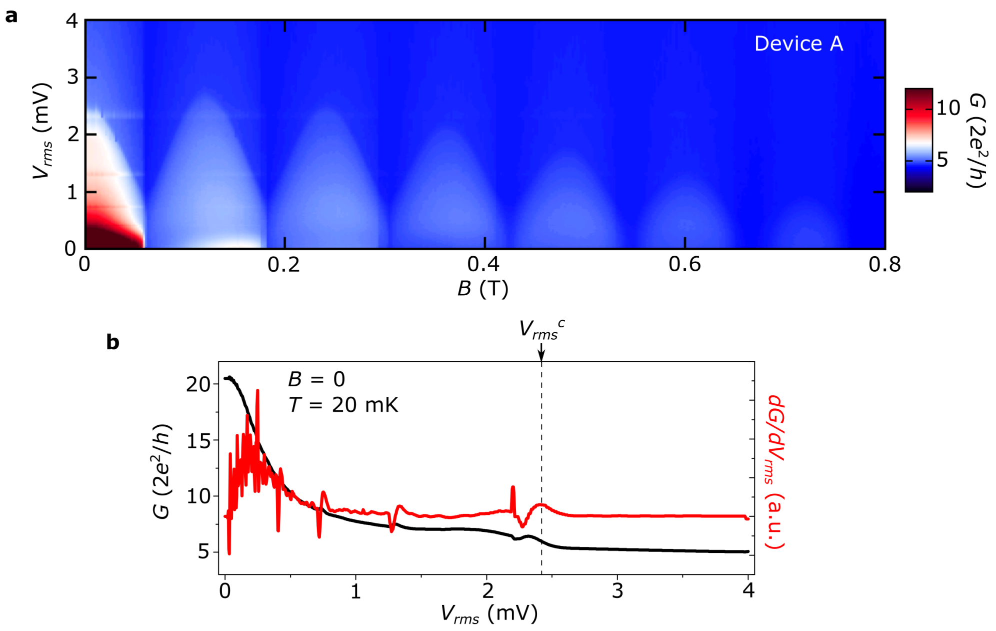

FIG. S4. **Data of AC signal heating without numerical differentiation**. **a**, same dataset as shown in Fig. 4d of the main text without numerical differentiation, i.e., zero-bias differential conductance, G, as a function of the nominal microwave signal amplitude, V_{rms}, and magnetic field for $f = 3.492$ GHz. **b**, Linecut of panel **a** at $B = 0$. The black and red lines show $G(V_{rms})$ and $dG/dV_{rms}(V_{rms})$, respectively. Heating in the island leads to a substantial drop of G. At a critical amplitude V_{rms}^c, the island turns normal, leading to the suppression of Josephson and Andreev processes.

S3. GATE DEPENDENCE OF CONDUCTANCE DIPS

Fig. S5 displays the dependence of the conductance dips as a function of the left junction side gate, $V_{g,L}$. All three dips move to higher voltages as the total device resistance increases. This behavior is similar to that observed in single Josephson junctions [1]. Notice that when $V_{dip,I} >> 2\Delta/e$, features related to the superconducting gap are clearly visible at low V.

We note that the main conclusions from our work are not impacted by any particular gate configuration, in particular if charging effects remain negligible. While Coulomb blockade and asymmetries in the resistances of the nanowire junctions may add further complexity to the heat transport, they are out of the scope of this work and will be studied in a more systematic manner in the future.

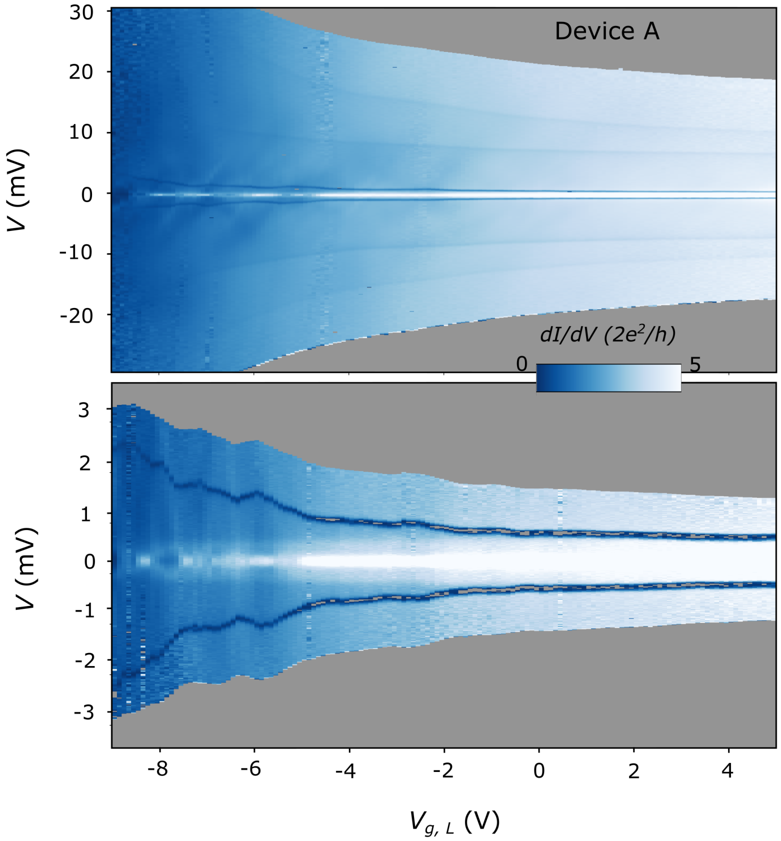

FIG. S5. **Gate dependence of high- and low-bias dips for device A**. $dI/dV(V)$ measurements as a function of $V_{g,L}$. Top panel, the three dI/dV dips disperse as the resistance of the left junction is tuned from the open regime (positive gate voltages) towards pinch-off. Note that I_{exs} decreases with the device conductance and, as a consequence, the contrast of the dip eventually goes down. This is particularly true for the high-bias dip in this dataset. Bottom panel, gate dependence of the low-bias dip. The lock-in excitation was fixed at 200 μV (5 μV) for the top (bottom) panel. Note that, for certain values of the gate voltage, the dip displays a negative differential conductance (NDC). In the color plot, the NDC features are seen in grey (also used for the parameter space with no experimental data, at higher voltages).

S4. OBTAINING DEVICE PARAMETERS BY FITTING LITTLE-PARKS OSCILLATIONS

The main results of this work refer to the dependences of the thermal dips as a function of magnetic field, which allow us to identify the dominant heat dissipation mechanisms for the distinct superconductors in our device. Here we provide a short description of the protocol that we have employed for fitting the experimental data, by taking into account the tuning of $T_{c,j}$ by the Little-Parks effect. In short, we employ a model of a thin cylinder in the dirty limit within the framework of Abrikosov-Gorkov theory [2, 3]. The corresponding evolution of T_c as a function of a parallel magnetic field is given by,

$$\ln\left(\frac{T_c(\alpha)}{T_c(0)}\right) = \Psi\left(\frac{1}{2}\right) - \Psi\left(\frac{1}{2} + \frac{\alpha}{2\pi k_B T_c(\alpha)}\right), \tag{1}$$

where Ψ is the digamma function, and α is the pair-breaking parameter. A small angle θ between the nanowire x-axis and the applied magnetic field is introduced as an additional fitting parameter, resulting in a parallel and a perpendicular contribution to the magnetic field: $B_{\parallel} = B\cos\theta$, $B_{\perp} = B\sin\theta$. Consequently, the total pair-breaking is given by $\alpha = \alpha_{\parallel} + \alpha_{\perp}$ [4, 5] with,

$$\alpha_{\parallel} = \frac{4{\xi_{\mathrm{S}}}^2 T_c(0)}{A}\left[\left(m - \frac{\Phi_{\parallel}}{\Phi_0}\right)^2 + \frac{t_{\mathrm{S}}^2}{d^2}\left(\frac{\Phi_{\parallel}^2}{\Phi_0^2} + \frac{m^2}{3}\right)\right], \tag{2}$$

$$\alpha_{\perp} = \frac{4{\xi_{\mathrm{S}}}^2 T_c(0)\lambda}{A}\frac{\Phi_{\perp}^2}{\Phi_0^2}. \tag{3}$$

Here m denotes the fluxoid quantum number, $\Phi_{\parallel} = B_{\parallel}A$, $\Phi_{\perp} = B_{\perp}A$, $A = \pi d^2/4$, and λ is a free fitting parameter determining the perpendicular contribution to pair-breaking.

In this work, we have fixed $t_S = 7.8$ nm and $\lambda = 1.7$. We then use three free fitting parameters, namely θ, B_p, and ξ_{S}, and Eqs. (1-3) to fit the experimental data to,

$$V_{dip}(B) = \zeta \cdot T_c(B), \tag{4}$$

$$V_{dip}(B) = \beta \cdot \sqrt{T_c(B)^n - T_{bath}^n}, \tag{5}$$

to discern between dissipation by quasiparticle diffusion (Eq. (4)) or electron-phonon coupling (Eq. (5)). More concretely, we numerically calculate $V_{dip}(B)$ from Eqs. (4) and (5), letting the AG fitting parameters vary, within reasonable limits, until a good correspondence

with the experimental data is achieved. As discussed in the main text, the former (latter) provides the best fit for the superconducting leads (island). A thorough discussion of the validity of fitting parameters is detailed in the Supplementary Material of our previous work [1]). In Figures S6 and S7, we plot the extracted values of $V_{dip,j}$ for devices A and B, respectively, along with $V_{dip}(B)$ curves obtained numerically using the procedure described above. For the low-bias dips, we include $V_{dip}(B)$ calculated with different values of n for comparison.

The values of the extracted parameters of the fits shown in the main text, as well as of other parameters of interest are summarized in the tables below for devices A and B. These values are in good agreement with estimates from previous works on similar material [1, 4, 5]. Note that the choice of the shell thickness stems from a careful characterization of the Little-Parks oscillations in several wires of the same batch, including in measurements where a rotation of the magnetic field was carried out, thus allowing to conclude that t_S cannot be thicker than $\sim$ 8 nm.

**Device A**						
**Superconductor**	$T_{bath}[K]$	$T_c(0)[K]$	ξ_S [nm]	$B_p[mT]$	$t_s[nm]$	θ [deg]
Lead R	0.02	1.29	95	119	7.8	2
Lead L	0.02	1.33	95	122	7.8	2
Island	0.02	1.43	80	121	7.8	2

**Device B**						
**Superconductor**	$T_{bath}[K]$	$T_c(0)[K]$	ξ_S [nm]	$B_p[mT]$	$t_s[nm]$	θ [deg]
Island	0.25	1.43	92	222	7.8	3

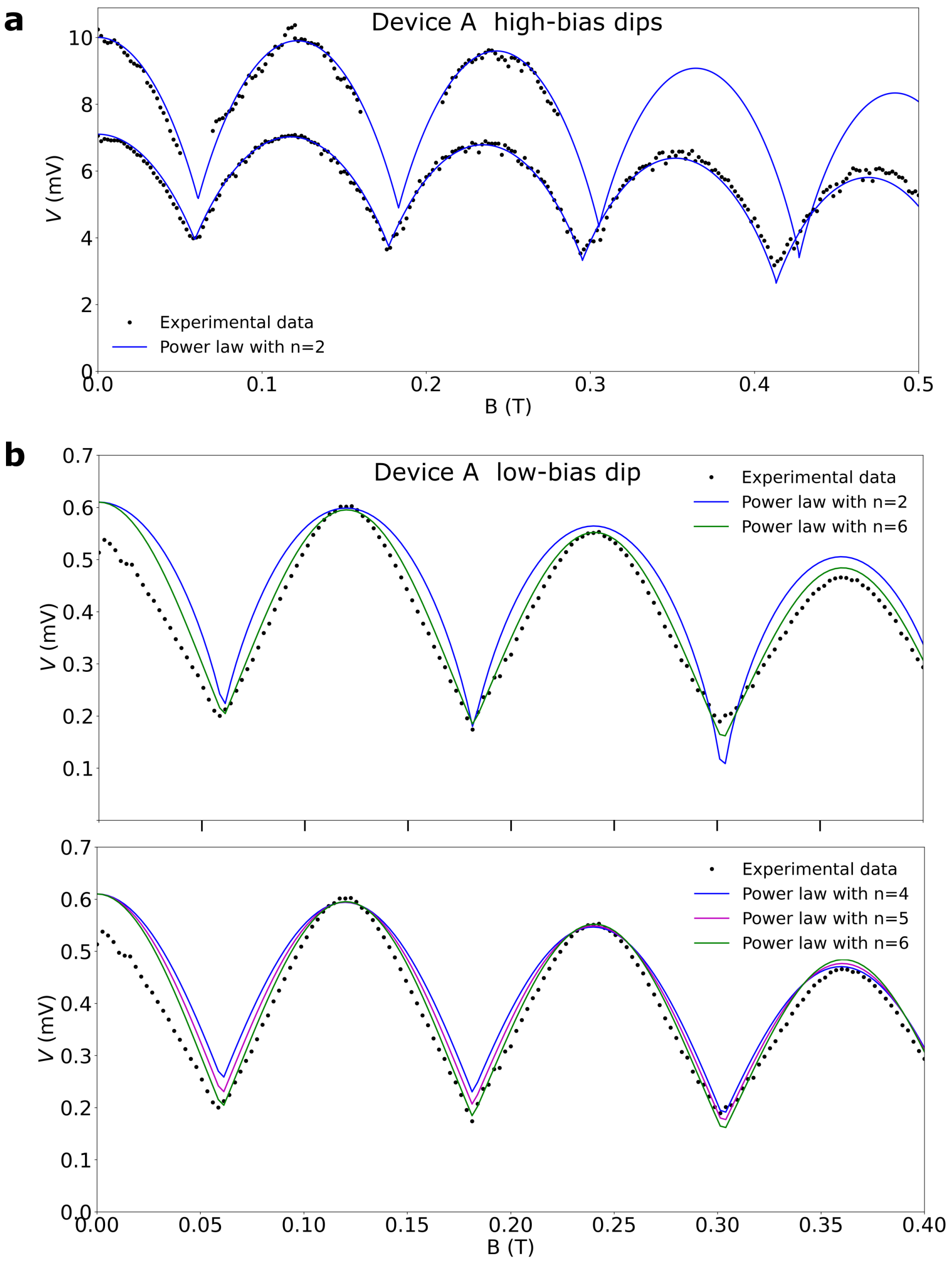

FIG. S6. **Fits to $V_{dip,j}(B)$ for device A.** **a**, Extracted high-bias dip positions (black dots) as a function of external magnetic field. The blue lines correspond to $V_{dip}(B)$ curves numerically calculated using Eq. (4). **b**, The low-bias dip position is shown as black dots. The solid lines are $V_{dip}(B)$ curves calculated using Eq. (5) for different values of n. Note that the AG parameters were optimized for each of the curves, attempting to get a good agreement with the experimental points. As mentioned in the main text, the zeroth lobe displays a lower $V_{dip,I}$ when compared to lobe 1. This is discussed further in section S5.B.

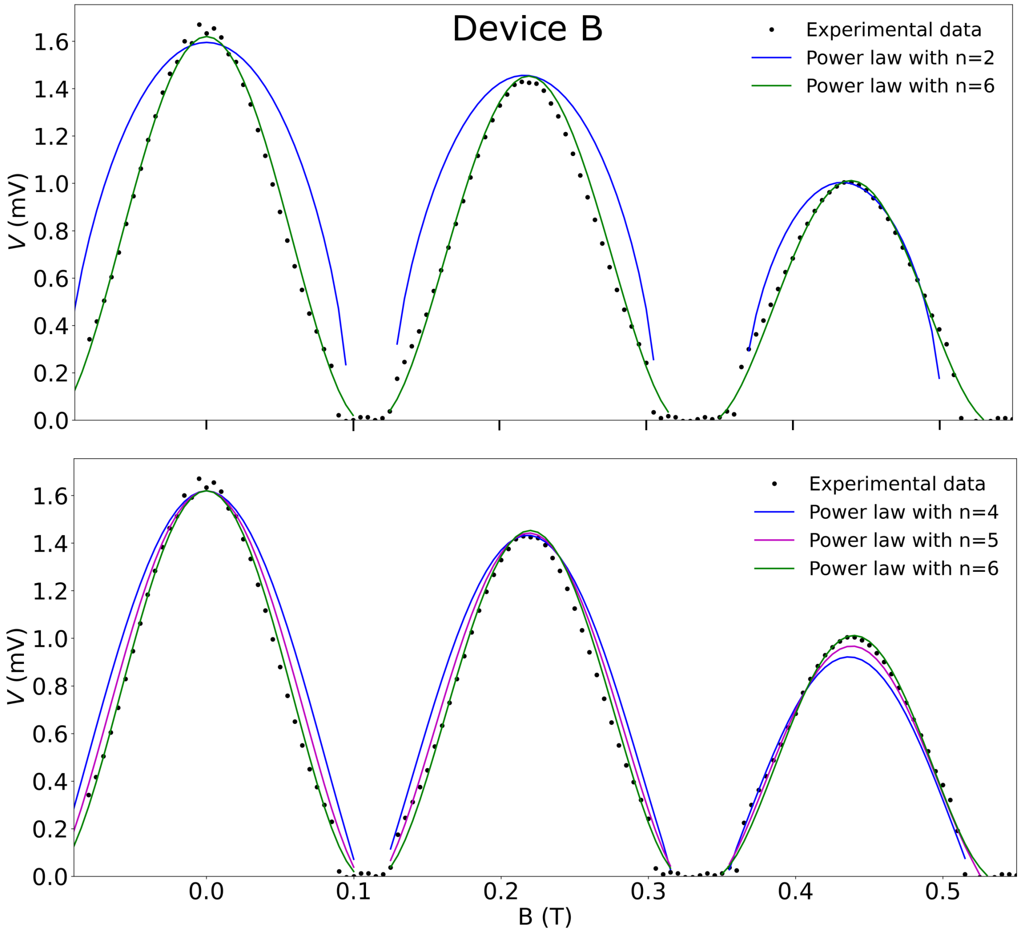

FIG. S7. **Fits to $V_{dip,j}(B)$ for device B**. Low-bias dip position (black dots) as a function of external magnetic field. The blue and green lines correspond to $V_{dip}(B)$ numerically calculated using Eq. (5) for $n = 2$ and 6, respectively. The lower panel shows the same experimental data as in the top, but with curves calculated for $n = 4$, 5 and 6. Note that the AG parameters were optimized for each of the curves, attempting to get a good agreement with the experimental points.

S5. TRANSPORT THEORY

In this section, we elaborate on the transport theory used in the main text to explain the position of dips and distribution of power between different parts of the device. To focus on the essential components necessary to explain the thermal dips, we choose a minimal assumptions approach to model the setup, compared to the more elaborate Floquet-Keldysh calculations presented in Ref. [1]. We start by assuming that the Island system can be modeled as a S-S-S junction, with a left, right, and island side denoted by L, R, and I respectively, and that the I-V and power on the L to I and on the I to R junction is given by a normal resistive term in addition to excess current terms, $I_{exs,j}$, from Andreev reflections on the three present gaps. Assuming current conservation we obtain,

$$I = \frac{V_L}{R_L} + I_{exs,L} + I_{exs,LI} = \frac{V_R}{R_R} + I_{exs,R} + I_{exs,RI}, \tag{6}$$

$$P_{h,L} = \frac{V_L^2}{2R_L} + V_L I_{exs,LI}, \qquad P_{h,R} = \frac{V_R^2}{2R_R} + V_R I_{exs,RI}, \tag{7}$$

$$P_{h,I} = \frac{V_L^2}{2R_L} + \frac{V_R^2}{2R_R} + V_L I_{exs,L} + V_R I_{exs,R}, \tag{8}$$

here $I_{exs,LI}$ is the excess current stemming from Andreev reflections of the island gap at the left junction, which for asymmetrical transmission and number of channels could be different than corresponding right-hand side, $I_{exs,RI}$. In the following, as of the symmetric gating and fabrication of the junctions, we assume $R_L \approx R_R \approx R/2$ and equal transmissions such that $I_{exs,LI} \approx I_{exs,LR} \approx I_{exs,I}$. With these assumptions we arrive at the main equations of the main text, Eqs. (2-4), where we neglected terms containing,

$$\Delta V = V_L - V_R = \frac{R}{2}\left(I_{exs,L} - I_{exs,R}\right) \tag{9}$$

which is valid for $|V| = |V_L + V_R| \gg |\Delta V|$. At small voltages, V, the assumption remains valid so far the junctions and left/right leads are largely symmetric, such that $I_{exs,L} \approx I_{exs,R}$. For larger voltages when a dip occurs, rendering e.g. $I_{exs,L} = 0$, ΔV is maximal, but lead dips occur typically at voltages $V \gg \frac{R}{2} I_{exs,L/R}$ again supporting the assumption.

Next, we comment on the validity of the model. First, in treating the island as a S-S-S system we assumed that there are no charging effects on the island, and that each element has a well-defined temperature and reaches thermal equilibrium between transport processes.

That charging is negligible is supported by the open gate setting, and, correspondingly, we do not observe Coulomb diamonds or Fabry-Perot lines in these regimes. Regarding thermalization of non-equilibrium distributions from transport processes, our experiment is not conclusive. A non-equilibrium distribution could close the gap, similar to a thermal distribution at T_c, and so distinguishing them in experiment requires an energy-resolved measurement [6]. The quality of fits with heating models of, e.g. magnetic field data in the main text, supports that local thermal equilibrium models describe the experiment well. Moreover, if R_L is significantly different from R_R unequal voltage drop would develop across the two junctions, resulting in more power going to the lead at the larger voltage drop, but not greatly affecting the amount of heat going to the island. As the current experiment focuses on the island heating we disregard such corrections. Finally, it is only for $|V| \gg 2\Delta$ that the current is well described as a normal part plus an excess part. For $|V| \lesssim \Delta$ the I-V is characterized by Multiple Andreev Processes [1], which saturates into a excess current at large bias. This results in non-linear I-V's on each junction, which to solve exactly requires a self-consistent calculation of V_L and V_R to enforce $I_L = I_R$. As we focus mainly on the dips, and not low bias structure, we instead choose to model the excess currents as,

$$I_{exs,j} = \frac{I_{exs}}{2}\frac{\Delta_j(T)}{\Delta}\tanh\left(\frac{AV}{\Delta_I(T)+\Delta_L(T)+\Delta_R(T)}\right) \tag{10}$$

with I_{exs} denoting the total measured excess current, $\Delta(T)/\Delta$ describing the closing of the gap with temperature using $\Delta = 0.2$ meV for Al, and the hyperbolic-tangent describing the onset of the excess current with bias. We choose $A = 10$ which yields a rapid onset of excess current, similar to what is observed in experiment. Lastly, in order to obtain $\Delta_j(T)$ we solve the regular self-consistent gap equation,

$$1 = \nu_{Al} U \int_{\Delta}^{\hbar\omega_D} d\omega \, \frac{1}{\sqrt{\omega^2 - \Delta_j(T)^2}} \tanh \frac{1}{2}\frac{\omega}{k_B T}, \tag{11}$$

with U being the interaction strength, ω_D the Debye frequency, and ν_{Al} the Fermi density of states. For all leads, we use numbers for Al.

A. Cooling mechanisms

In this subsection, we describe in more detail the cooling of the leads through thermally excited quasiparticles, and discuss other possible cooling channels. Following the derivations

of Ref. [1, 7], we assume that an InAs-Al lead of normal resistance $R_{lead,j}$ is contacted to a metallic lead anchored to the bath temperature, T_{bath}. Solving for the temperature gradient that satisfies thermal equilibrium of the lead yields the following integral equation for the cooling power at the junction interface at temperature, T_j,

$$P_{c,j} = \frac{8k_B^2}{e^2 R_{lead,j}} \int_{T_{bath}}^{T_j} dT\, T \int_{\frac{\Delta_j(T)}{2k_B T}}^{\infty} dx\, \frac{x^2}{\cosh^2 x}, \tag{12}$$

which, if inverted, can be used to express T_j as a function of $P_{c,j}$. The solution yields a rapid increase of T_j for low $P_{c,j}$, as a finite number of quasiparticles needs to be excited to transport the heat, but converges towards the metallic Wiedemann-Franz result for large $P_{c,j}$. Specifically at $T_j = T_{c,j}$, at which a thermal dip occurs, the relation can be simplified to Eq. (5) of the main text with,

$$\Lambda(T_{bath}) \approx 2.112 \left[1 - \left(\frac{T_{bath}}{T_c}\right)^{\gamma}\right], \tag{13}$$

with $\gamma \approx 3.6$. This expression is used to fit thermal dips of the leads. However, the lower extracted values of the exponent $\gamma = 2.4$ and $\gamma = 2.8$ (see Fig. S1) instead of the theoretical expected value from Eq. (13) show that the temperature dependence is somewhat sensitive to device details, for instance, the length of the leads. With these equations, we can self-consistently solve for the I-V and temperatures of the leads and island. First, one calculates Eqs. (2-4) of the main text to obtain the power going into each element, then one finds the temperatures via Eq. (6) for Island and Eq. (S12) for the leads, and use them to obtain $\Delta_j(T_j)$ which in turn modifies Eqs. (2-4). This loop is then iterated till convergence is obtained. A simulated I-V is shown in Fig S8 with parameters chosen to match Fig. 1d of the main text. Apart, from detailed behavior of the low-bias structure, the simulation and experiment match well. Regarding asymmetry of high-bias dips, corresponding to the right plot of Fig. S8, this can either be explained by differences of R_j or $R_{lead,j}$, with $j = L, R$, as,

$$\frac{V_{dip,L}}{V_{dip,R}} = \sqrt{\frac{R_R R_{lead,R}}{R_L R_{lead,L}}}, \tag{14}$$

which follows from eq. (5) in the main text and Eqs. (6-8) assuming $I_{exs,j} \ll V_j/R_j$, appropriate for high-bias. Consequently, from the observed high-bias peaks the asymmetry of R_L and R_R cannot be deduced without prior knowledge of $R_{lead,L}$ and $R_{lead,R}$.

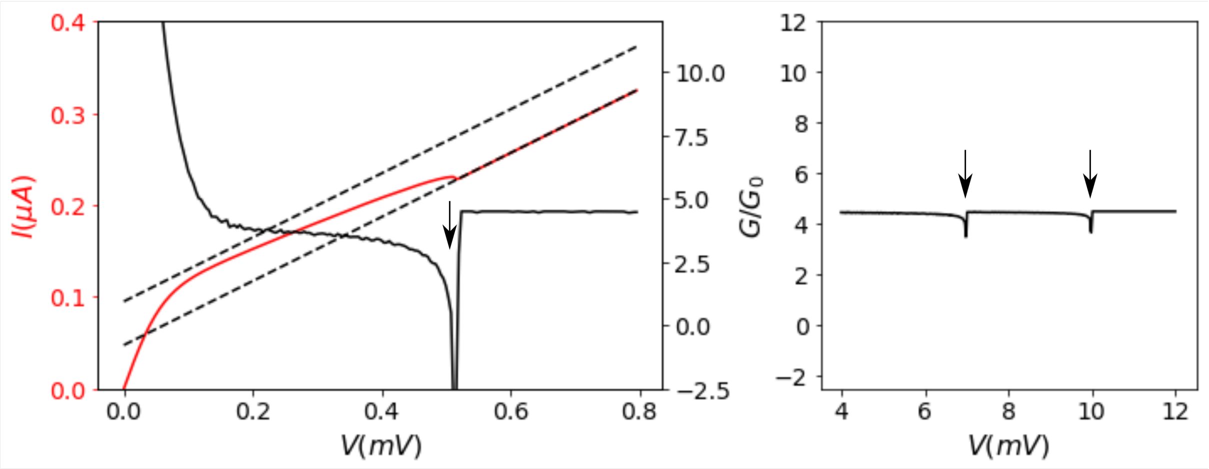

FIG. S8. **Simulation of device A low and high bias dips.** Theory simulations with parameters chosen to correspond with Fig. 1d of the main text. Parameters are; $G = 4.5\ G_0$, $I_{exs} = 0.095\ \mu$A, $T_{bath} = 0.25$ K, $R_{lead,L} = 1.8\ \Omega$, $R_{lead,R} = 0.9\ \Omega$, and $\Sigma = 4.7\times10^9\ \frac{\mathrm{W}}{\mathrm{m^3K^6}}$. The critical temperature is set to $T_c = 1.4$ K for all components. Temperatures, T_j, with $j \in L, R, I$ are obtained by self-consistently solving Eqs. (2-4) and (6) from the main text, and Eq. (S12).

Next, we discuss another potential source of cooling stemming from thermal gradients across the junction. For a single metallic N-N junction the Wiedemann-Franz relation yields,

$$P_{WF,j} = \frac{\pi^2 k_B^2}{6e^2 R_j}\left(T_I^2 - T_j^2\right), \tag{15}$$

which is added to the heating power of the lead and subtracted for the island. Due to the higher temperature of the island, this term will carry heat from the Island to the leads. Consequently, at the low bias dip for device B, where the system is all metallic, assuming the leads to be at bath temperature we obtain the following cooling for the island,

$$P_{WF,I} = \frac{\pi^2 k_B^2}{6e^2}\frac{R_L + R_R}{R_L R_R}\left(T_c^2 - T_{bath}^2\right) \approx \frac{2\pi^2 k_B^2}{3e^2 R}\left(T_c^2 - T_{bath}^2\right) \approx 12\ \text{pW}, \tag{16}$$

which is 8% of the $P_{dip,I} \approx 150$ pW measured in experiment. Although this term has a different T_c dependence, and would therefore contribute to the fits of the Little-Parks oscillations, it is too small to be observed. To note, if the asymmetry of R_L and R_R is increased this contribution grows. By fitting Little-Park we found that this term could be no larger than 30%, otherwise the fit quality diminishes, supporting our assumption of relatively symmetric junctions and most of the cooling stemming from phonon coupling. Assuming metallic leads for device A we obtain $P_{WF,I} = 33$ pW, which is about 50% of the

measured $P_{dip,I} \approx 60$ pW. However, as the leads are superconducting for device A we expect a greatly reduced thermal conductance due to the gap, so the 33 pW serves as an upper bound.

B. Discussion of device A lobe 0 discrepancy

For device A a noticeable decrease of $V_{dip,I}$ occurs between lobe 0, centered at zero magnetic field, and center of lobe 1 of the Little-Park oscillations, as highlighted in Fig. S10a-b. This is surprising as for our current estimation of Little-Park parameters, superconductivity, and therefore also T_c, should be close to fully restored at the center of lobe 1. Thus, equal $V_{dip,I}$ is expected. Interestingly, a significant decrease of the overall excess current is observed between lobe 0 and lobe 1, which in Fig. S10a-b can be seen as a reduction of the zero-bias conductance. At the center of lobe 0 we fit the I-V and obtain $I_{exs} \approx 0.095$ μA, while at lobe 1 we instead find $I_{exs} \approx 0.02$ μA.

For a larger ratio of I_{exs}/I the critical temperature would be reached at a lower V_{dip} and a higher I_{dip}, assuming constant cooling power, P_{dip}, as can be seen from Eqs. (3) in the main text. In Fig. S10c we use self-consistent calculations to show this effect for device A, where approximately 25% of the measured current is excess current for lobe 0 and only about 5% for lobe 1. Qualitatively, the increase of $V_{dip,I}$ of about $0.05-0.1$ mV and a slight decrease of $I_{dip,I}$ is captured by the model, although the model slightly overestimates the increase of V_{dip} and underestimates the decrease of I_{dip}. We attribute these discrepancies to the imperfect modeling of low-bias I-V, which is also apparent in the $I_{dip,I} \approx 0.23$ μA compared to the measured value of ~ 0.3 μA. Lastly, we comment that the curves in Fig. S10c do not keep the $I_{dip,I}V_{dip,I}$ product constant, although the cooling power is $P_{dip,I}$ is kept constant. This is due to the asymmetric distribution of power in the excess current, with proportionally more power going to the island than the leads for higher I_{exs}.

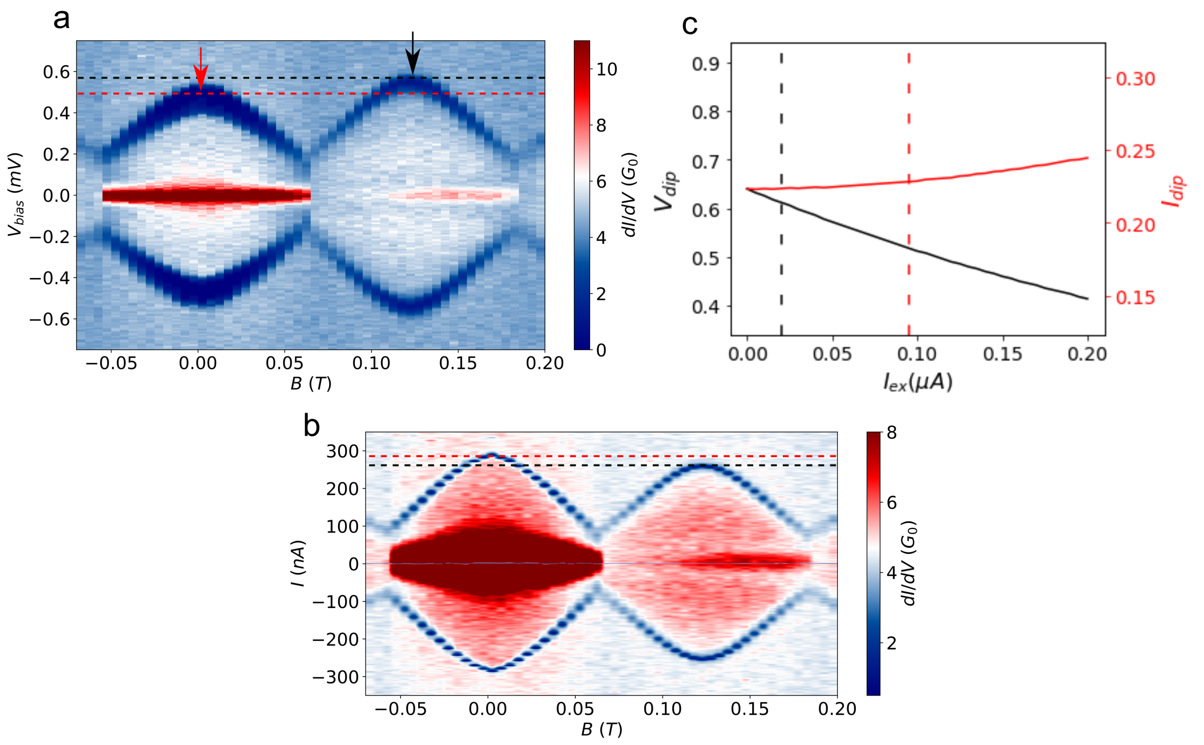

FIG. S9. **Comparisons of lobe 0 and lobe 1.** Low-bias conductance measurements of $V_{dip,I}$ shown as a function of voltage (**a**) and current (**b**) for lobe 0 and lobe 1, similar to Fig. 2b of the main text. **c** Self-consistent solutions of $V_{dip,I}$ and $I_{dip,I}$ using the same parameters as in Fig. S8, apart from the varying I_{exs}. The red dashed line indicates the lobe 0 solution and the black the lobe 1 solution.

C. Effects of Microwave

We now turn to the application of a microwave signal on this system, and we assume that it results in an AC voltage signal in addition to the applied DC voltage, yielding a net voltage, $V(t) = V_{dc} + V_{ac}\sin(\omega_0 t)$, with ω_0 denoting the microwave frequency. In order to establish the response of the system it is important to consider the thermalization time scales. Let us consider the heat-balance equation for the superconducting island,

$$\mathcal{C}_e \frac{dT}{dt} = P_{h,I} - U\Sigma\left(T_I^n - T_{bath}^n\right) \tag{17}$$

with $\mathcal{C}_e$ indicating the heat-capacity of the Island which for a metal is given by $\mathcal{C}_e = \frac{\pi^2}{3}k_B^2\nu_{Al}UT_I$. Assuming $P_{h,I}$ to be constant we find an exponential decay to the steady-state solution, $dT/dt = 0.0$, with a characteristic time scale of Eq. (7) in the main text, which for device A is about $\tau_{th} \approx 10$ ns. Here we note that if the Island is superconducting then the electronic heat capacity would be exponentially suppressed by a factor $\sim e^{-\Delta_I/k_BT_I}$, but the electron-phonon coupling, Σ, is expected to be similarly suppressed by a $e^{-\Delta_I/k_BT_I}$ factor, which would therefore cancel out in Eq. (7). However, as we are primarily interested in the vicinity of the island dip, for which the island is metallic or close to, we expect τ_{th} to be the appropriate time scale.

Next, to compare with experimental results, we detail how to simulate in the limit of slow AC compared to thermalization, $\omega_0 \ll 1/\tau_{th}$, or fast $\omega_0 \gg 1/\tau_{th}$. In the slow limit, the system reaches thermal steady-state for each value of $V(t)$, and correspondingly the long-time averaged I-V curve can be obtained as a convolution of the self-consistently calculated DC response, $I_{DC}(V)$, yielding,

$$I(V_{dc}, V_{ac}) = \int_{-\infty}^{\infty} dv\; I_{dc}(V_{dc} - v)F(v, V_{ac}), \tag{18}$$

with the probability of being at voltage, v, in the AC oscillation captured by $F(v, V_{ac}) = 1/\mathrm{Re}\sqrt{V_{ac}^2 - v^2}$. As this function is peaked at $\pm V_{ac}$ the effect of the convolution is to split peaks present in the DC into two as AC increases.

In the opposite limit, the system goes through multiple AC oscillations before thermally stabilizing, meaning that the temperatures, T_j, remain approximately constant across a single period. Here we fix T_j, and so also Δ_j, and evaluate the average current and power across a cycle,

$$\langle I(t)\rangle = \frac{1}{T_p}\int_0^{T_p} dt\; I(t), \quad \langle P(t)\rangle = \frac{1}{T_p}\int_0^{T_p} dt\; P(t), \tag{19}$$

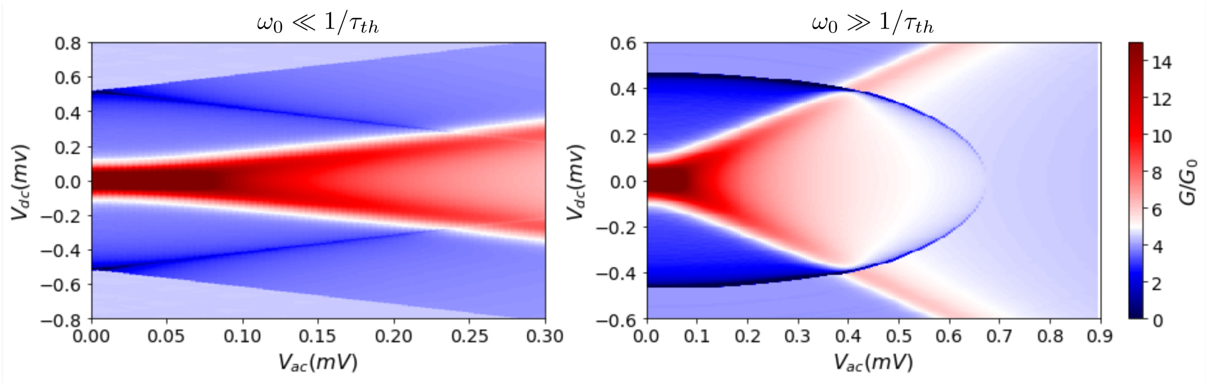

FIG. S10. **Comparison of slow and fast AC.** Simulations of differential conductance as a function DC and AC bias in the low bias regime, focusing on the island dip, as to compare with Fig. 4c of the main text. Parameters used are identical to those in Fig. S8. The main finding is the dip peak splitting in the slow regime while closing in the fast, similar to measurement. For both cases, the inner conductance peak splits into two branches.

using Eqs. (2-4). These average powers are then used as $P_{h,j}$ to obtain new T_j's until self-consistence has been reached. This has the primary effect of introducing a heating term, $V_{ac}^2/2R$, divided as the DC across the two leads and the Island, which as V_{ac} increases gradually lowers the thermal dips until they cross zero V_{dc}. This is seen in Fig. 4b of the main text which is computed using identical parameters as Fig. S8. Unlike, the slow regime the dips do not split in this case as a thermal dip only occurs at one given temperature.

---

[1] A. Ibabe, M. Gómez, G. O. Steffensen, T. Kanne, J. Nygård, A. L. Yeyati, and E. J. H. Lee, Joule spectroscopy of hybrid superconductor–semiconductor nanodevices, Nat. Commun. **14**, 1 (2023).

[2] A. A. Abrikosov and L. P. Gor'kov, Contribution to the theory of superconducting alloys with paramagnetic impurities, Zh. Eksp. Teor. Fiz **39**, 1781 (1960).

[3] S. Skalski, O. Betbeder-Matibet, and P. R. Weiss, Properties of Superconducting Alloys Containing Paramagnetic Impurities, Phys. Rev. **136**, A1500 (1964).

[4] A. Vekris, J. C. Estrada Saldaña, J. de Bruijckere, S. Lorić, T. Kanne, M. Marnauza, D. Olsteins, J. Nygård, and K. Grove-Rasmussen, Asymmetric Little–Parks oscillations in full shell double nanowires, Sci. Rep. **11**, 1 (2021).

[5] S. Vaitiekėnas, P. Krogstrup, and C. M. Marcus, Anomalous metallic phase in tunable destructive superconductors, Phys. Rev. B **101**, 060507 (2020).

[6] H. Pothier, S. Guéron, N. O. Birge, D. Esteve, and M. H. Devoret, Energy Distribution Function of Quasiparticles in Mesoscopic Wires, Physical Review Letters **79**, 3490 (1997).

[7] M. Tomi, M. R. Samatov, A. S. Vasenko, A. Laitinen, P. Hakonen, and D. S. Golubev, Joule heating effects in high-transparency Josephson junctions, Phys. Rev. B **104**, 134513 (2021).
